# Room‐Temperature Direct Homolysis of C_sp3_─H Bond via Catalyst‐Free Photoexcitation

**DOI:** 10.1002/EXP.20240237

**Published:** 2025-04-10

**Authors:** Qi Miao, Meng Liu, Jun Wang, Pan Wu, Changjun Liu, Jian He, Giacomo Lo Zupone, Wei Jiang

**Affiliations:** ^1^ Low‐Carbon Technology and Chemical Reaction Engineering Laboratory School of Chemical Engineering Sichuan University Chengdu P.R. China; ^2^ State Key Laboratory of Environmental‐Friendly Energy Materials School of Materials and Chemistry Southwest University of Science and Technology Mianyang China; ^3^ Department of Mechanics Mathematics and Management Polytechnic of Bari Bari Italy

**Keywords:** catalyst‐free, C─H direct homolysis, photochemistry

## Abstract

The C─H bond is the most abundant chemical bond in organic compounds. Therefore, the development of the more direct methods for C─H bond cleavage and the elucidation of their mechanisms will provide an important theoretical basis for achieving more efficient C─H functionalization and target molecule construction. In this study, the catalyst‐free photon‐induced direct homolysis of C_sp3_─H bonds at room temperature was discovered for the first time. The applicable substrate scope of this phenomenon is very wide, expanding from the initial benzyl compounds to aliphatic alcohols, alkanes, olefins, polymers containing benzyl hydrogens, and even gaseous methane. Experiments and calculations have demonstrated that this process involves rapid vibrational relaxation on the femtosecond time scale, leading to the formation of hydrogen radical and carbon radical. Importantly, the direct homolysis of C_sp3_─H bonds is independent of the presence of oxidants, highlighting its spontaneous nature. Additionally, the cleaved hydrogen radical exhibits diverse reactivity, including coupling reactions to produce hydrogen gas (H_2_), reduction of oxygen to generate hydrogen peroxide (H_2_O_2_), and reduction of carbon dioxide to formic acid (HCOOH). Notably, in the field of H_2_O_2_ production, the absence of a catalyst allows for the bypassing of inherent drawbacks associated with photocatalysts, thereby presenting significant potential for practical application. Furthermore, the cleaved carbon radicals display enhanced reactivity, providing excellent opportunities for direct functionalization, thereby enabling efficient C─H bond activation and molecular construction. Overall, this significant discovery offers a valuable new strategy for the production of bulk chemicals, organic synthesis, low‐carbon and hydrogen energy industries, as well as environmental treatment.

## Graphical abstract

In the absence of catalysts and additives, under only ambient temperature and light conditions, organic compounds undergo vibrational relaxation processes after photon excitation, followed by direct C‐H cleavage to generate hydrogen radicals and carbon radicals. The substrate scope is broad, ranging from benzylic substrates to alcohols, alkenes, alkanes, benzylic hydrogen‐containing polymers, and even gaseous methane.

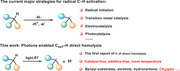

## 1 Introduction

C─H bonds are arguably the most abundant chemical bonds in organic compounds [1], and the radical C─H activation [2] has been recognized a reliable method to accelerate the development of direct molecular construction with high step‐ and atom‐ economy [3, 4], garnering widespread attention from chemists. Currently, strategies are primarily focused on radical initiation [5, 6, 7, 8], transition metal catalysis [9, 10, 11], anodic oxidation [12, 13], and photocatalysis [2, 14, 15, 16, 17] to enable the departure of hydrogen radical or pairs of hydrogen protons and electron, generating highly reactive carbon radicals for subsequent functionalization. Notably, while transition metals can offer precise control over selectivity, they are often toxic or overly expensive, and may require ligands to enhance activity. Electrocatalytic technologies face limitations such as high costs and durability concerns; photocatalysis, despite offering a more sustainable solution, typically requires costly catalysts and grapples with inherent defects like the recombination of photogenerated electrons and holes, hindering further practical application. Therefore, the development of more direct methods for C─H bonds cleavage, along with the elucidation of the mechanisms proposed, will offer an important theoretical basis for achieving more efficient C─H activation and target molecules construction, and will also signify significant implications for the further advancement of organic chemistry.

In this field, our work has made significant progress. We disclosed the first catalyst‐free direct homolysis of C_sp3_–H bonds at room temperature, utilizing high‐energy photons to achieve a process that is both direct and environmentally benign. Our approach avoids the need for any catalysts or external radical initiators, simplifying the reaction setup and broadening the range of substrates. Specifically, we demonstrate the generation of H_2_ in toluene under xenon lamp irradiation in a nitrogen atmosphere, as confirmed by gas chromatography (GC) and high‐resolution mass spectrometry (HRMS) analysis. The detection of 1,2‐diphenylethane in the toluene solution (**HRMS**; *m/z* (ESI) calculated for [C_14_H_14_+H^+^]^+^ = 183.1168, found = 183.1159) (Figure S2) further supports the occurrence of C_sp3_‐H homolysis. This finding is not restricted to toluene; we also observe H_2_ formation in ethylbenzene, butylbenzene and cumene (Figure S3), suggesting that C_sp3_‐H bond homolysis may be universal under these conditions.

The implications of our discovery are far‐reaching. If confirmed as a general phenomenon, photon‐enabled catalyst‐free direct homolysis of C_sp3_–H bonds could offer a more sustainable, direct, and efficient method for C‐H bond activation, potentially revolutionizing the field of chemistry. This method has the potential to reduce reliance on precious metals, minimize side reactions, and enhance the overall atom economy of synthetic routes. Therefore, a comprehensive and in‐depth investigation into the mechanism of high‐energy photon enabled catalyst‐free direct homolysis of C_sp3_–H bonds is imperative, encompassing the potential reaction pathways, intermediates and transition states, etc.

In this study, a series of mechanistic experiments and characterization investigations are carried out using toluene as a model substrate to provide conclusive evidence for the existence of a catalyst‐free, photon‐enabled direct C_sp3_–H bond homolysis mechanism. Using state‐of‐the‐art electron paramagnetic resonance (EPR), femtosecond transient absorption spectroscopy (fs‐TAS) and Gaussian simulation calculation, the potential transition states, intermediates and reaction processes involved in this mechanism have been comprehensively investigated and confirmed. Once the reaction mechanism has been determined, a systematic exploration of the substrate scope for this C_sp3_–H bonds homolysis phenomenon will be carried out to assess the potential implications and prospects in the field of chemistry, catalysis, and environmental treatment.

## 2 Results and Discussion

### 2.1 Confirmatory Experiments

**FIGURE 1:**
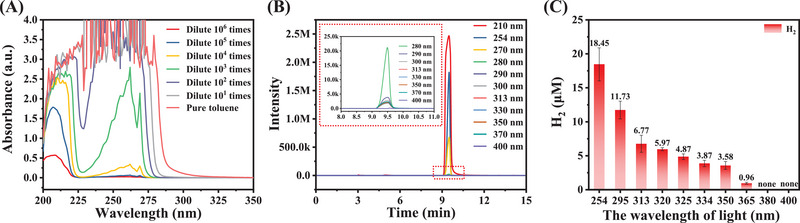
Confirmatory experiments: (A) UV–Vis absorption spectra of toluene with different concentrations; (B) HPLC retention time of toluene at different monitoring wavelengths; (C) The assessments on the homolysis of toluene benzylic C_sp3_–H under different wavelengths.

Light absorption is a prerequisite for photochemical reactions. Notably, the absorption spectrum of toluene is primarily centered around 210 nm, with almost no absorption peaks are observed beyond 300 nm (Figure 1A). However, our experiments demonstrating H_2_ production (Figure S1) indicate that toluene can absorb photons from a Xe lamp within the range of 300–1000 nm and become photoexcited. Meticulous analysis reveals a progressive redshift in the absorption peak of toluene with increasing concentration, suggesting the possible formation of molecular aggregates, which exhibit a gradual redshift in their absorption peaks [18, 19, 20, 21]. This implies that the reaction system is capable of absorbing photons and undergoing reactions.

To further confirm that toluene solutions at low concentrations can also absorb photons within the range of 300–1000 nm, we conducted validation experiments using high‐performance liquid chromatography (HPLC) to measure the absorbance of significantly diluted toluene solutions (10^5) at various wavelengths (Figure 1B). These experiments revealed that toluene molecules exhibit absorption within the 220–370 nm range, consistent with the UV‐Vis spectrum of pure toluene. This indicates that toluene, even at low concentrations, can absorb photons (λ = 300–370 nm) and subsequently be photoexcited.

Subsequently, an assessment on the homolysis of toluene benzylic C_sp3_–H under different wavelength lights was performed, using H_2_ production as the quantitative criterion. As shown in Figure 1C, the maximumH_2_ production was observed at 254 nm. As the wavelength increases, the production of H_2_ gradually decreases, reaching zero at wavelengths exceeding 370 nm, which is consistent with the absorption spectrum of toluene. When toluene was irradiated under visible light (λ ≥ 420 nm), no H_2_ was detected, even overnight at 60°C. This can be attributed to the weaker energy of longer‐wavelength photons, which cannot be absorbed to effectively excite toluene and trigger the C_sp3_–H direct homolysis. Therefore, only high‐energy photons play a crucial role in the direct homolysis of the benzylic C_sp3_–H in toluene, with higher photon energy leading to easier excitation even under ambient conditions [22].

### 2.2 Mechanism

**FIGURE 2:**
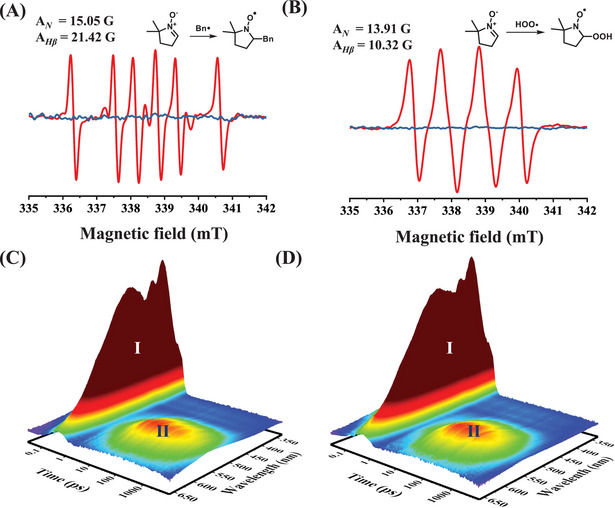
Schematic of the proposed hydrogen radical protocol for H_2_O_2_ photosynthesis. EPR measurement of toluene irradiated by Xe lamp in an oxygen‐free nitrogen atmosphere (A) and an oxygen atmosphere (B), the red line indicates the EPR signals obtained under Xe lamp, whereas the blue line represents the EPR signals from the dark control experiments; fs‐TAS spectrum of toluene in an oxygen‐free nitrogen atmosphere (C) and an oxygen atmosphere (D).

The electron paramagnetic resonance (EPR) analysis using DMPO was conducted to intuitively elucidate the radical formation and the occurrence of benzylic C_sp3_–H direct homolysis in toluene after photoexcitation. As shown in Figure 2A, in the absence of oxygen, the detected EPR signals of toluene irradiated by Xe lamp (λ = 300–1000 nm) were attributed to benzylic radical (**Bn•**), which exhibited the characteristic hyperfine splitting constants of the six‐wire signal (A*_N_ =* 15.05 G, A*_Hβ_* = 21.42 G). However, no signal corresponding to **H•** was observed. This could be attributed to the significantly higher intensity of the **Bn•** and DMPO adducts compared to that of the **H•**. Fortunately, high‐resolution mass spectroscopy (**HRMS**; *m/z* (ESI) calculated for [C_6_H_12_NO•+Na^+^]^+^ = 137.0811, found = 137.0806) detected the adduct of **H•** and DMPO (Figure S4).

Additionally, when 2,2,6,6‐Tetramethylpiperidinooxy (**TEMPO**) was introduced into the reaction system, the presence of adduct of **H•** and **TEMPO**, **TEMPOH**, was confirmed (**HRMS**; *m/z* (ESI) calculated for [C_9_H_19_NO+H^+^]^+^ = 158.1539, found = 158.1545) (Figure S5), and adduct of **Bn•** and **TEMPO**, **TEMPOBn** was also confirmed (**HRMS**; *m/z* (ESI) calculated for [C_16_H_25_NO+H^+^]^+^ = 248.2009, found = 248.2008) (Figure S6). These findings further confirmed the presence of **H•** and **Bn•**. Interestingly, when exposed to the atmospheric environment, the detection of a peroxy radical (**HOO•**) signal (Figure 2B, A*_N_* = 13.91 G, A*_Hβ_* = 10.32 G) by EPR suggests that homolytic **H•** may enable oxygen reduction reaction (ORR), leading to the generation of **HOO•** and H_2_O_2_.

To gain further insight into the behavior of toluene after photoexcitation for the initiation of direct homolysis and radical formation, fs‐TAS was performed under an oxygen‐free nitrogen atmosphere (Figure 2C). The excited state of toluene initially underwent a vibrational relaxation process on the femtosecond time scale corresponding to the region **I** after photoexcitation. Subsequently, the benzylic C_sp3_–H of toluene underwent homolytic cleavage to generate **H•** and **Bn•** corresponding to the region **II**. When the absorbed energy greatly exceeds the bond dissociation energy, direct C‐H homolysis occurs, while homolysis is nearly absent when the energy is below the bond dissociation energy (Figure S7) [23]. Kinetic analysis of **II** revealed the existence of three kinetic processes. Two mainly reversible processes (Figures 2C, S8, and S9, approximately 2.87 ps) of those, which can be attributed to the formation and recombination of **Bn•** and **H•** resulting from the homolysis of the C─H bond. Combining the results from Figure S2, the third process is speculated to be the generation of 1,2‐diphenylethane by **Bn•** coupling (Figures 2C and S8, approximately 2.87 ps).

Previous studies have indicated that the cleavage of C─H bonds typically requires the participation of hydrogen abstracting agents such as excited‐state oxygen [24, 25, 26], radical intermediates [27, 28], or excited‐state carbonyl compounds [29], etc. To further investigate the effect of these hydrogen abstracting agents on the cleavage of benzylic C─H bonds in toluene, the fs‐TAS on toluene bubbled with oxygen was performed. Oxygen, rather than other reagents, was chosen as the hydrogen extraction agent because it does not directly interfere with the spectral absorption behaviour of toluene, thus allowing more accurate observation and study of the cleavage behaviour of the benzyl C─H bond in toluene. As shown in Figure 2D, under oxygen conditions, the photoexcited toluene also underwent a vibrational relaxation process at the femtosecond level, that is, region **I**. There was a new peak (**II**) around 550 nm same as Figure 2C, Kinetic analysis of **II** also revealed the existence of three kinetic processes (Figures 2D and S10), with two mainly reversible processes exhibiting equal timescales of approximately 2.96 ps. It was inferred that this corresponded to the benzylic C─H homolysis of toluene, resulting in the generation of **Bn•** and **H•**, as well as the recombination. Notably, the homolysis rate of benzylic C─H shows negligible difference under both oxygen and oxygen‐free conditions (2.96 ps vs 2.87 ps), suggesting that the homolytic cleavage process of benzylic C─H is independent of oxygen and occurs spontaneously. Another process would be the formation of 1,2‐diphenylethane, estimated to occur within a timescale of approximately 2.96 ps (Figure S10).

Both H_2_ production experiments (Figure S1), EPR (Figure 2A) and HRMS (Figures S4 and S6) confirmed the presence of **H•**. The fs‐TAS (Figures 2C,D and S8–S10) demonstrated the presence of the direct homolysis mechanism of C─H bonds after toluene is photoexcited. This homolysis mechanism is independent and not influenced by the presence of oxidizing species in the environment. However, it is necessary to further elucidate the process of C─H homolysis after toluene photoexcitation using Gaussian calculations.

**FIGURE 3:**
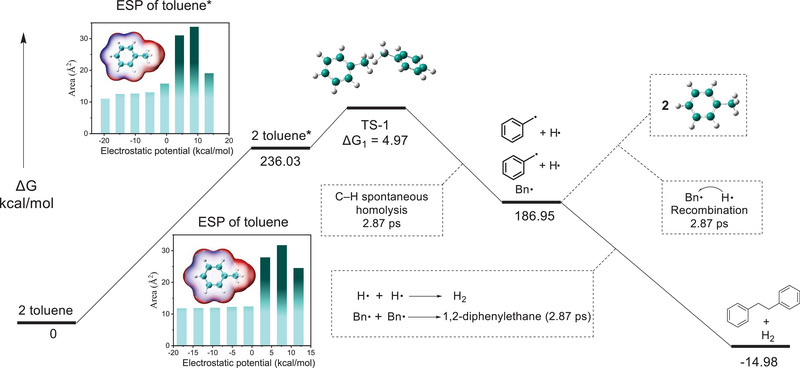
Computational study of the potential reaction pathways of the toluene benzylic C─H bond direct homolysis using B3LYP‐D3/6‐311++G(d,p) calculation conditions.

The change in the molecular surface electrostatic potential (ESP) of toluene has been investigated^31^, as shown in Figure 3. Photoexcited toluene demonstrates a discernible distribution of electrostatic potential across its molecular surface, characterized by the benzene ring displaying predominantly negative charges, while the methyl group carries predominantly positive charges. The positive charges are notably localized within the range of 4.13‐13.63 kcal/mol, encompassing a cumulative area of 82.78 Å^2^. Conversely, the negative charges are distributed more uniformly across the surface, encompassing a cumulative area of 64.95 Å^2^. These findings suggest that photoexcited toluene possesses polarity and the benzylic hydrogen demonstrates high reactivity. The following frontiers orbital calculations also proved it (Table S1). After excitation, the excited toluene undergoes a low energy barrier (*ΔG_1_* = 4.97 kcal/mol) to reach transition state TS‐1, indicating the facile direct homolysis of the benzylic C─H bond in the excited state. Combined with the fs‐TAS results, it is suggested that the photon may transfer energy to the benzylic C─H bond via a vibrational relaxation process [30, 31], leading to the direct homolysis and generation of **Bn•** and **H•**. The produced **Bn•** and **H•** may undergo coupling to form 1,2‐diphenylethane and H_2_, or recombination to reform toluene.

The discussion and computational evidence above confirmed that the toluene benzylic C─H bond direct homolysis upon exposure to light at room temperature, resulting in the formation of **H•** and **Bn•** (carbon radicals). Further investigations are necessary to carried out to explore the substrate scope of this phenomenon, enabling a comprehensive understanding and potential applications.

### 2.3 Substrate Scope

The production of H_2_ can indirectly provide evidence for the existence of direct C─H bond homolysis after photoexcitation. As shown in Table 1, almost all benzylic compounds can produce H_2_ after photoexcitation. For instance, toluene produces hydrogen gas and 1,2‐diphenylethane after 5 h of Xe lamp irradiation. It is speculated that upon photoexcitation, toluene undergoes vibrational relaxation process at the femtosecond level after photoexcitation, leading to the generation of **H•** and **Bn•**. The **H•** couple to form H_2_, while the **Bn•** couple to form 1,2‐diphenylethane (Figure S2). This phenomenon is observed in other benzyl alkanes (**1‐9**) as well.

**TABLE 1:** Substrate scope for photosynthesizing H_2_.

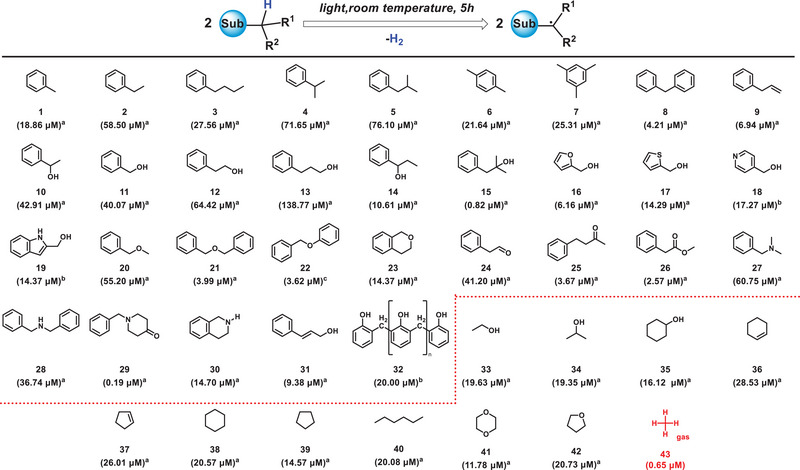

*Note*: ^a^Liquid compounds directly participate in the reaction; ^b^Solid, water‐soluble compounds use water as a solvent; ^c^Solid, water‐insoluble compounds use stable solvents like benzene to prevent reactive hydrogen interference. The Xe lamp is used for sufficient photon energy; otherwise, a mercury lamp is selected. The standard curve for H_2_ (Figure S23).

^a^Reaction of compounds (5 mL), ^b^compounds (1 g) mixed with H_2_O (5 mL) or ^c^compounds (1 g) mixed with benzene (5 mL) under N_2_ at 20°C for 5 h. 1 mL of gas was taken and fed into gas chromatography (FULI GC9790‐II) for analysis, and the GC is equipped with a thermal conductive detector (TCD), 5A Molecular Sieve column, using Argon (99.999%) gas as carrier gas. Only **38–41, 43** use Mercury lamp (λ = 254–365 nm, light intensity = 75 mW·cm^−2^), the rest use Xe lamp (λ = 300–1000 nm, light intensity = 320 mW·cm^−2^).

Benzyl alcohols (**10‐19**) can also produce H_2_ after photoexcitation, indicating the occurrence of benzylic C─H bond direct homolysis. Taking benzyl alcohol (**11**) as an example, the detection of hydrobenzoin, diphenylacetaldehyde, and benzaldehyde in the reaction solution of benzyl alcohol indicates a possible cleavage of the C─H bond in compound **11**, leading to the formation of benzyl radicals that may couple to generate hydrobenzoin. Subsequently, this intermediate undergoes dehydrogenation to yield benzoin (Figure S10) or rearrangement to produce diphenylacetaldehyde (Figure S11). Additionally, the benzyl radicals derived from **11** can further undergo dehydrogenation to form benzaldehyde. Apart from benzyl alcohol, benzyl ethers (**20‐23**) may also exhibit the behaviour of direct homolysis of benzyl C─H after photons irradiation. Taking benzyl methyl ether (**20**) and isochroman (**23**) as examples, the benzyl radical of **20** directly C–C coupled after photoexcitation (Figure S13), whereas the radical intermediate of the C─H direct homolysis of **23** undergoes dissociation of **H•** to form 1H‐2‐Benzopyran (Figure S14).

Benzyl aldehydes and benzyl amines can also photosynthesize H_2_ after photoexcitation. Taking phenylacetaldehyde (**24**) and N,N‐dimethylbenzylamine (**27**) as examples, upon irradiation of **24**, the generation of 2,3‐diphenylsuccinaldehyde (Figure S15) was observed, which might be attributed to the coupling of benzyl radicals generated by the C─H homolysis. A similar phenomenon is also observed in **27**, wherein H_2_ is produced along with the formation of product of C–C coupling (Figure S16). In benzylamine substrates, photoexcited tetrahydroisoquinoline (**30**) undergoes direct homolysis of the benzylic C─H bond, followed by multi‐step dehydrogenation to generate quinoline derivatives (Figures S17 and S18). Apart from benzyl substrates, allyl compounds, phenol‐formaldehyde resins and a wide range of aliphatic alkanes (**33‐42**) also exhibit the phenomenon of H_2_ generation after photoexcitation. As shown in Figure S19, H_2_, cyclohexane and 1‐Hexene are observed after the photoexcitation of n‐hexane (**40**), suggesting the possibility of direct homolysis of the C─H bond to generate **H•** and n‐hexane carbon radical, followed by the coupling of **H•** to form H_2_ and the coupling of n‐hexane radicals to form cyclohexane.

Excitingly, gaseous methane, under room temperature and atmospheric pressure, requires only exposure to ultraviolet light to produce H_2_ in the reaction system. The amounts of H_2_ produced after irradiation for 5, 10, and 24 h are 0.65, 1.08, and 1.24 µM, respectively. This suggests that methane undergoes direct C─H bond homolysis upon absorption of high‐energy photons, unlike the conventional industrial process of high‐temperature thermal catalytic cracking [32], providing a new pathway for methane utilization and conversion. These phenomena highlight the universality of direct C─H bond homolysis in organic molecules under light irradiation.

When the N_2_ atmosphere is switched to O_2_, the **H•** generated from the C─H bond homolysis of the above compounds can reduce oxygen to produce H_2_O_2_. As shown in Table 2, all benzyl substrates can photosynthesize H_2_O_2_ in O_2_ atmosphere. Notably, benzyl methyl ether (**20**), isochroman (**23**), and N,N‐dimethylbenzylamine (**27**) demonstrated high photochemical efficiency in the generation of hydrogen peroxide, with 4‐h yields amounting to 311.54 mM, 610.08 mM, and 244.40 mM, respectively, far beyond the photocatalytic system [33, 34, 35, 36, 37, 38, 39, 40, 41, 42]. Furthermore, the green hydrogenation technology, based on water as the hydrogen source [43, 44] and Pd_1_‐mpg‐C_3_N_4_ [45, 46, 47] as the catalyst, can achieve the photon‐enabled, eco‐friendly closed‐loop production of H_2_O_2_ using benzyl alcohol as an intermediate (Figure S20). The discovery offers new insights for the development of photosynthesizing H_2_O_2_.

**TABLE 2:** Substrate scope for photosynthesizing H_2_O_2_.

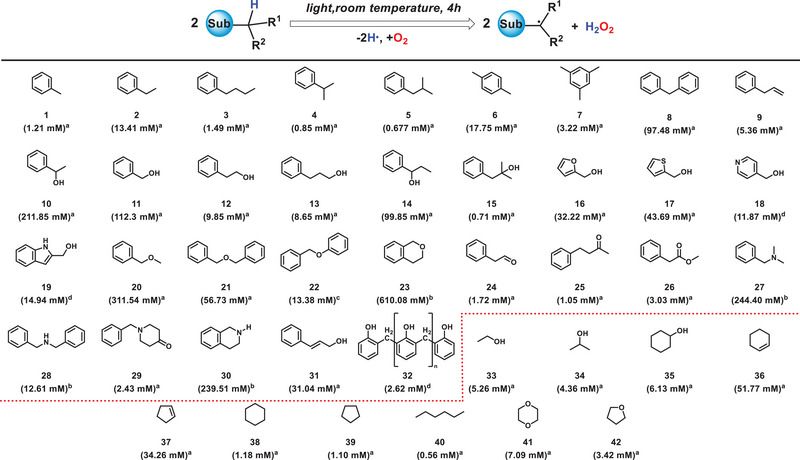

*Note*: ^a and b^Liquid compounds directly participate in the reaction; ^c^Solid, water‐insoluble compounds use stable solvents like benzene to prevent reactive hydrogen interference; ^d^Solid, water‐soluble compounds use water as a solvent. The standard curve for H_2_O_2_ (Figure S24).

^a^Reaction of compounds (5 mL) mixed with H_2_O (25 mL) under O_2_ (1 atm) at 20°C for 4 h

^b^Reaction of compounds (5 mL) under O_2_ (1 atm) at 20°C for 4 h

^c^Reaction of compounds (1 g) mixed with benzene (5 mL) under O_2_ (1 atm) at 20°C for 4 h

^d^Reaction of compounds (1 g) mixed with H_2_O (5 mL) under O_2_ (1 atm) at 20°C for 4 h, irradiated with a Xe lamp (λ = 300–1000 nm, light intensity = 320 mW·cm^−2^).

When the atmosphere is further switched from O_2_ to CO_2_, **H•** also can reduce CO_2_ to formic acid (Figures S21 and S22). As shown in Table 3, almost all the compounds can achieve such reduction, including typically inert compounds such as n‐hexane (**38**) and cyclohexane (**40**), which reach conversion rates of 85.89 µM and 89.31 µM, respectively. Notably, the conversion rate for benzyl methyl ether (**20**) is as high as 1153.29 µM. These results clearly demonstrate the high efficiency of this reduction process, even for inert compounds.

**TABLE 3:** Substrate scope for reducing CO_2_ to formic acid.

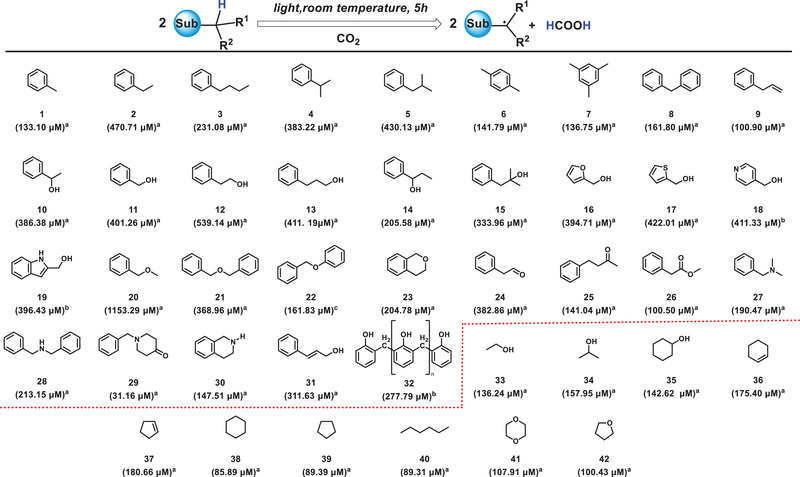

*Note*: ^a^Liquid compounds directly participate in the reaction; ^b^Solid, water‐soluble compounds use water as a solvent; ^c^Solid, water‐insoluble compounds use stable solvents like benzene to prevent reactive hydrogen interference. The Xe lamp is used for sufficient photon energy; otherwise, a mercury lamp is selected. The standard curve for formate (Figure S25).

^a^Reaction of compounds (5 mL), ^b^compounds (1 g) mixed with H_2_O (5 mL) or ^c^compounds (1 g) mixed with benzene (5 mL) under CO_2_ at 20°C for 5 h. Only **38–41** use mercury lamp (λ = 254–365 nm, light intensity = 75 mW·cm^−2^), the rest use xenon lamp (λ = 300–1000 nm, light intensity = 320 mW·cm^−2^).

## 3 Conclusion

In conclusion, we have found the first room‐temperature direct homolysis of C_sp3_–H via catalyst‐free photoexcitation. The scope of applicable substrates is remarkably broad, encompassing not only benzyl compounds but also extending to aliphatic alcohols, alkanes, olefins, polymers containing benzyl hydrogens, and even gaseous methane. The fs‐TAS and Gaussian calculations have demonstrated that this process involves vibrational relaxation on the femtosecond time scale, resulting in the formation of **H•** and carbon radical. Notably, the direct homolysis of C_sp3_–H bond remains unaffected by the presence of oxidants, indicating its spontaneous nature. These cleaved **H•** exhibit the ability to couple and generate H_2_, reduce O_2_ to produce H_2_O_2_, and reduce CO_2_ to HCOOH. This technology thus offers novel approaches and methods for catalyst‐free CO_2_ capture and H_2_O_2_ production. Importantly, the absence of a photocatalyst in this process circumvents the inherent limitations associated with photocatalysts, highlighting its practical potential. On the other hand, the cleaved carbon radical exhibit enhanced reactivity and offer the potential for direct functionalization, thereby facilitating the synthesis of target products.

Furthermore, H_2_O_2_ produced via this strategy allows for small‐scale localized production, utilizing solar energy for green synthesis. Coupled with wastewater treatment processes based on H_2_O_2_, this approach enables rapid, straightforward, and cost‐effective wastewater treatment.

In summary, this discovery presents a novel strategy for the production of bulk chemicals, organic synthesis, low‐carbon and hydrogen energy industries, and environmental remediation.

## Conflicts of Interest

The authors declare no conflicts of interest.

## Supporting information



Supporting Information

## Data Availability

Data supporting the results of this study are available from the corresponding author upon reasonable request.
