# Supplementary material for: Room‐Temperature Direct Homolysis of Csp3─H Bond via Catalyst‐Free Photoexcitation
Source: Exploration (Beijing). 2025 Apr 10;5(4):e20240237. doi: 10.1002/EXP.20240237 (PMC12380056; doi:10.1002/EXP.20240237)
Supplement: Supplementary file 1 — Supporting Information [file EXP2-5-e20240237-s001.doc]

Room-temperature Direct Homolysis of C_sp3_–H Bond via Catalyst-free Photoexcitation

*Qi Miao*^†^*, Meng Liu*^†^*, Jun Wang*^††^*, Pan Wu*^†^*, Chang-jun Liu*^†^*, Jian He*^†^*,* *Giacomo Lo Zupone*^†††^*, Wei Jiang** ^†^

^†^Low-carbon Technology and Chemical Reaction Engineering Laboratory, School of Chemical Engineering, Sichuan University, Chengdu, 610065 (PR China)

^††^State Key Laboratory of Environmental-Friendly Energy Materials, School of Materials and Chemistry, Southwest University of Science and Technology, Mianyang, 621010 (China)

^†††^Department of Mechanics, Mathematics and Management, Polytechnic of Bari, Via Edoardo Orabona 4, 70126 Bari, Italy

**Number of pages: 34**

**Number of tables: 1**

**Number of figures: 25**

**Number of Methods:8**

# Table of Contents

| **General Remarks** | S3 |
| --- | --- |
| **Methods** | S4-S9 |
| **Figure S1** Toluene Photosynthesize H_2_ (5 h) (natural sunlight irradiation on November 20, 2023) | S10 |
| **Figure S2** The cross-coupling reaction in toluene | S11 |
| **Figure S3** Benzene alkanes photosynthesize H_2_ (5 h) | S12-S13 |
| **Figure S4** The adduct of the **H•** and **DMPO** | S14 |
| **Figure S5** The adducts of the **H•** and **TEMPO** | S15 |
| **Figure S6** The adducts of the **Bn•** and **TEMPO** | S15 |
| **Figure S7** Femtosecond transient spectra of ethyl acetate in nitrogen gas | S16 |
| **Figure S8** The Kinetic fitting results of **II** under oxygen-free conditions at 550 nm | S17 |
| **Figure S9** The processes of formation and recombination of **Bn•** and **H•** | S17 |
| **Figure S10** The Kinetic fitting results of **II** under oxygen conditions at 564.6 nm | S18 |
| **Table S1** Calculation conditions of B3LYP-D3/6-311++G(d,p) and experimentally obtained electron orbital transition energies and absorption wavelengths of toluene | S19 |
| **Figure S11** The formation of benzoin | S20 |
| **Figure S12** The formation of diphenylacetaldehyde | S21 |
| **Figure S13** The formation of 1,2-dimethoxy-1,2-diphenylethane | S22 |
| **Figure S14** The formation of 1H-2-Benzopyran | S23 |
| **Figure S15** The formation of 2,3-diphenylsuccinaldehyde | S24 |
| **Figure S16** The formation of N1,N1,N2,N2-tetramethyl-1,2-diphenylethane-1,2-diamine | S25 |
| **Figure S17** The formation of 3,4-dihydroisoquinoline | S26 |
| **Figure S18** The formation of isoquinoline | S27 |
| **Figure S19** The formation of cyclohexane & 1-Hexene | S28 |
| **Figure S20** Construction of a closed-loop process | S29 |
| **Figure S21** The **^1^H NMR** of **H•** reduce CO_2_ to form HCOOH | S30 |
| **Figure S22** The **^13^C NMR** of **H•** reduce CO_2_ to form HCOOH | S30 |
| **Figure S23** The standard curve for H_2_ | S31 |
| **Figure S24** The standard curve for H_2_O_2_ | S32 |
| **Figure S25** The standard curve for Formate | S33 |
| **References** | S34 |

# General Remarks

All commercially available compounds were purchased from Aladdin, Shao-yuan, Bidepharm, Acros, Alfa and Adamas-beta. 1-phenylethanol (PA) (98%), benzyl Alcohol (99%), phenethyl alcohol (99%), 3-phenyl-1-propanol (98%), 1-phenyl-1-propanol (97%), 1,4-benzenedimethanol (98%), 4-biphenylmethanol (98%), 2-methyl-1-phenyl-2-propanol (98%), 2-phenyl-2-propanol (98%), furfuryl alcohol (99%), 2-thiophenemethanol (98%), benzyl ether (99%), benzyl phenyl ether (98%), isochroman (98%), 2,3-dihydrobenzofuran (98%), diphenylmethane (99%), allylbenzene (98%), benzylacetone (98%), 4-methoxyphenylacetone (98%), 4-phenylcyclohexanone (98%), 2-indanone(99%), methyl phenylacetate (99%), methyl phenylacetate (99%), N,N-dimethylbenzylamine (98%), dibenzylamine (99%), 1-benzyl-4-piperidone (99%), N-benzylacetamide (99%), 1,2,3,4-tetrahydroisoquinoline (98%), acetophenone (99%,GC) ,toluene (99.5%), ethylbenzene (99%), butylbenzene (99%), isopropylbenzene (99%), isobutylbenzene (99%), p-Xylene (99%), mesitylene (99%), allylbenzene (98%), cyclohexene (99%), cyclopentene (98%), cyclohexane (99.9%), cyclopentane (97%), hexane (97%), 1,4-dioxane (99.7%), tetrahydrofuran (99.9%) and cinnamyl alcohol (98%) were purchased from Adamas-beta. 4-hydroxymethylpyridine (98%) and 1H-indole-2-methanol (97%) were purchased from Bidepharm. benzyl methyl ether (95%) and phenol-formaldehyde resin (BR) were purchased from Aladdin. phenylacetaldehyde (95%) was purchased from Acros. Benzylamine (98.5%) and N,N-dimethylformamide (99.5%) were purchased from Alfa and Shao-yuan respectively. 5,5-Dimethyl-1-pyrroline-N-oxide (EPR radical capture reagent) was purchased from DOJINDO. Notably, the liquid substrate requiring reaction and the reaction solvent have been subjected to re-distillation before use, while solid substrates are used after simple column chromatography.

**Methods:**

**Photoreaction experiments:**

The H_2_ production experiment in the study was conducted using a quartz reaction tube (55 mL-Φ24*130 mm). The volume of the head space was 50 mL (**Fig. M1**). The experimental details for H_2_ production are as follows: 5 mL of reaction solution was added to the quartz reaction tube. Nitrogen gas was then blown into the tube for 30 minutes to remove oxygen and other gases, and a nitrogen atmosphere was then maintained. Then, the reaction solution was irradiated with 300 W Xe lamp (λ = 300–1000 nm) for 5 hours.


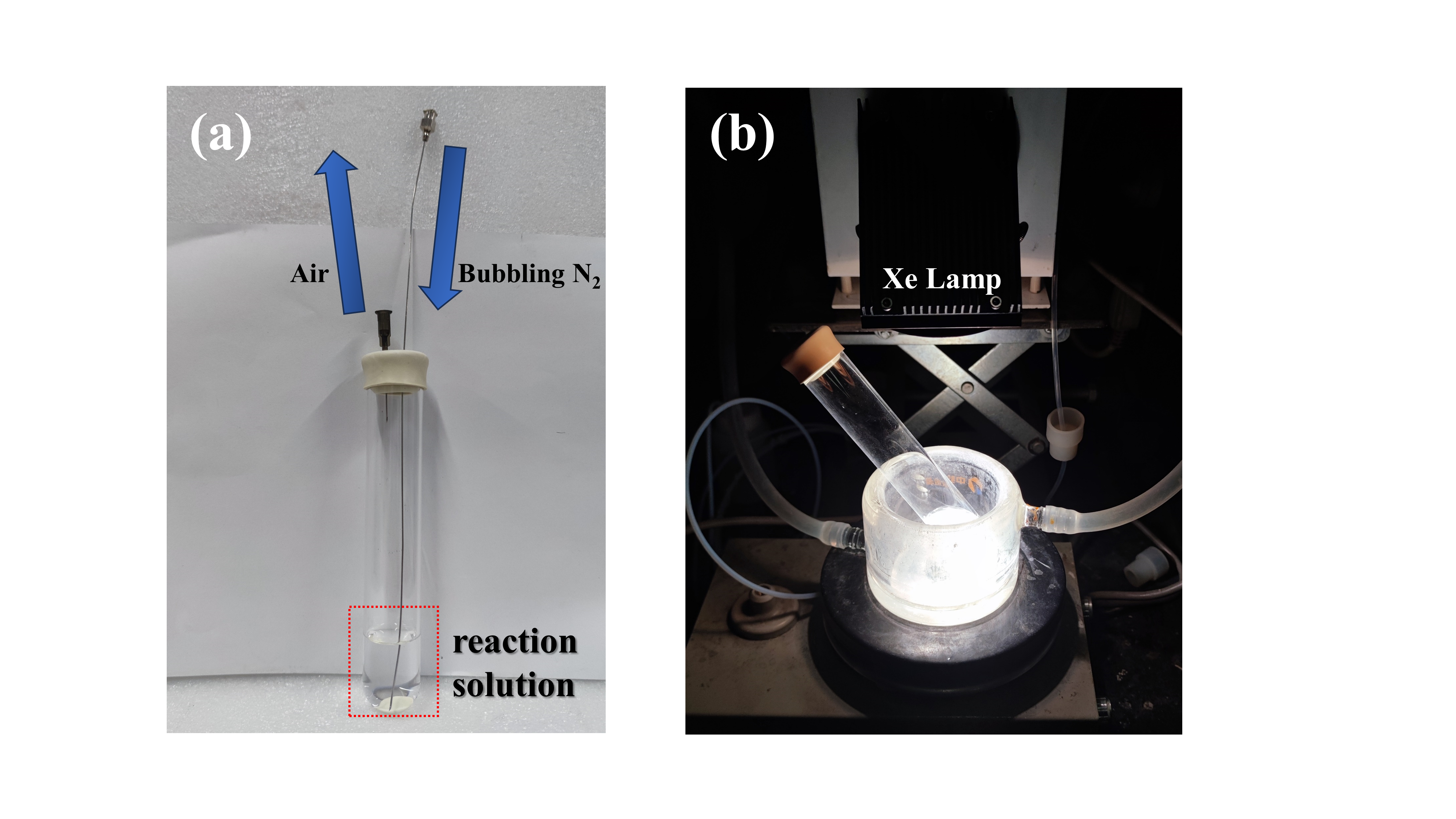


**Supplementary Fig. M1** a) Oxygen in the tube was removed by bubbling with nitrogen gas; b) The reaction solution was irradiated by Xe lamp

The H_2_O_2_ production experiment in the study was conducted using a quartz reaction reactor (220 mL-Φ5*120 mm) (**Fig. M2**). The reaction solution was stirred and continuously bubbled with oxygen/air for 15 minutes in the dark to exclude other gas. Subsequently, a 300 W Xe lamp (λ = 300–1000 nm) was used as the light source to drive the photoreaction. The reaction solution was collected and analyzed at arbitrary time intervals, and the H_2_O_2_ concentration was determined by the colorimetric method, specifically, the H_2_O_2_ solution to be measured (1.4mL), 3M H_2_SO_4_ 1.6mL mixed with 0.02M titanium potassium oxalate solution, shaking well, placed for 10 minutes, and then measured the absorbance with the UV-1500PC UV-visible Spectrophotometer of the Shanghai Meiyan Instrument Co.


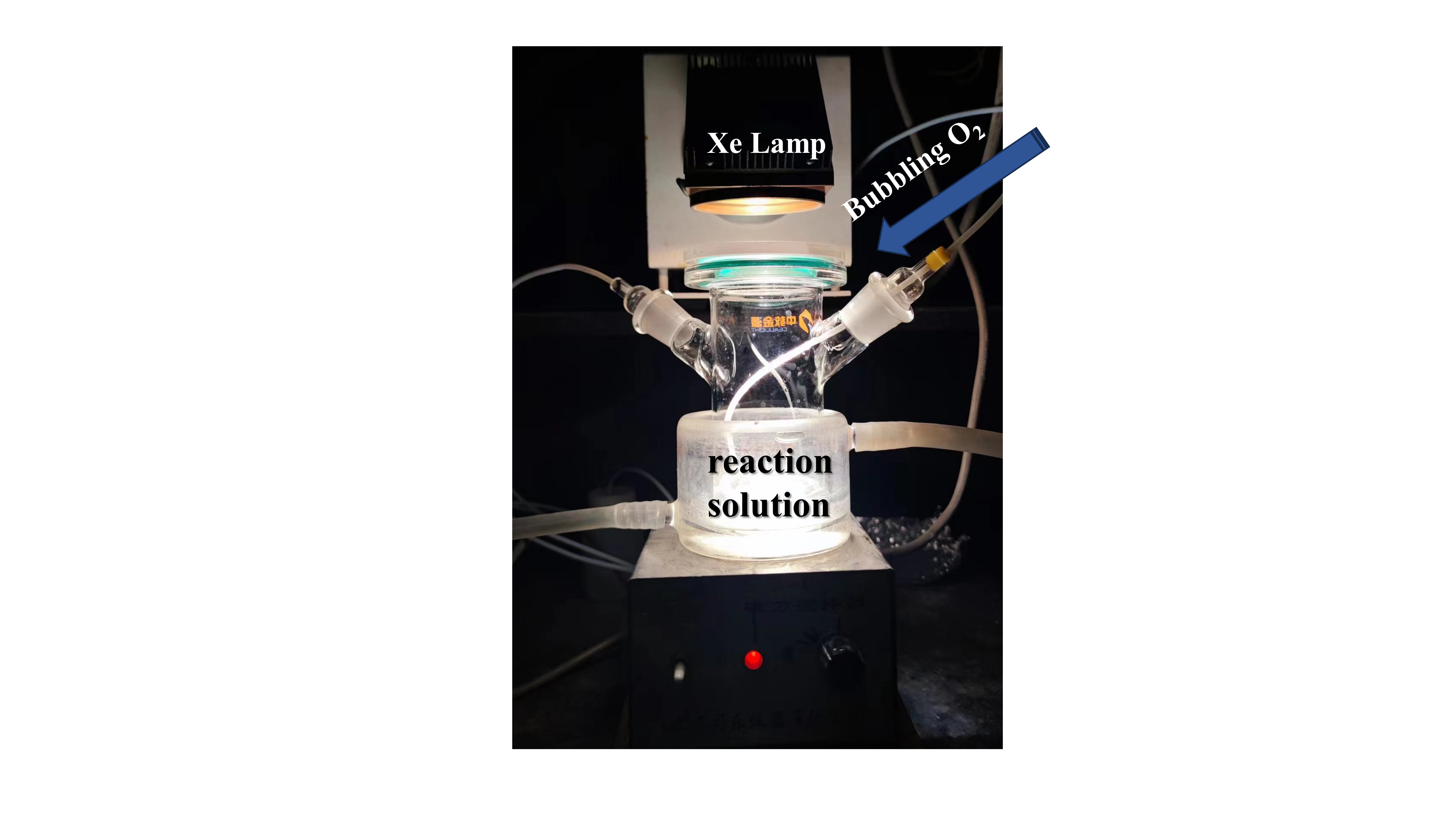


**Supplementary Fig. M2** The equipment for photosynthesizing H_2_O_2_ in an oxygen atmosphere.

The CO_2_ reduction to formic acid in the study was conducted using a quartz reaction tube (55 mL-Φ24*130 mm) (**Fig. M3**). The experimental details for CO_2_ reduction are as follows: 5 mL of reaction solution was added to the quartz reaction tube. CO_2_ was then blown into the tube for 30 minutes to remove oxygen and other gases, and a CO_2_ atmosphere was then maintained. Then, the reaction solution was irradiated with light for 5 hours.


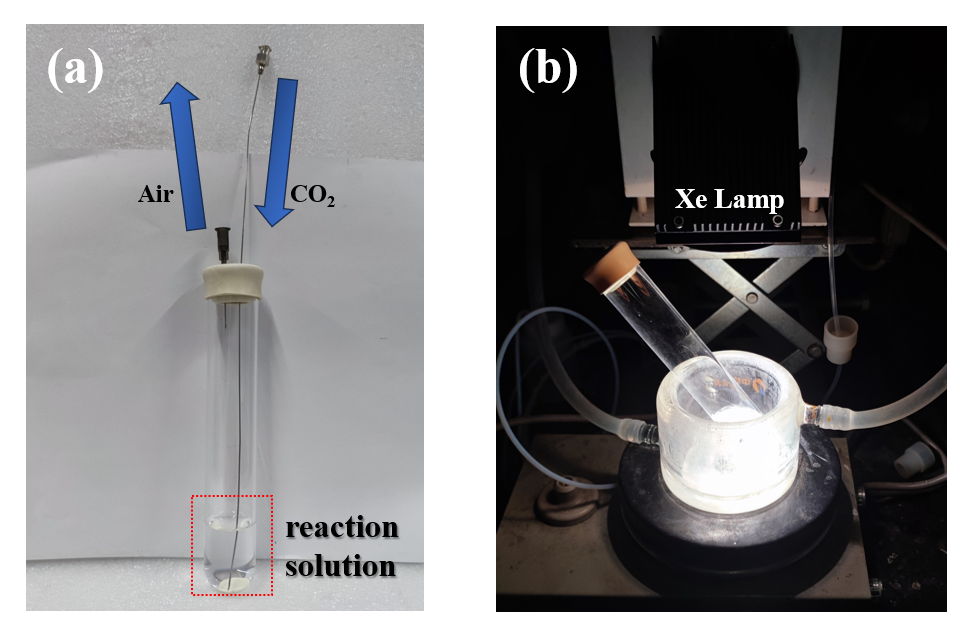


**Supplementary Fig. M3** a) Oxygen in the tube was removed by bubbling with CO_2_; b) The reaction solution was irradiated by Xe lamp

**Electron paramagnanetic resonance:**

The electron paramagnetic resonance (EPR) measurement was performed on a Minoscope MS-5000X instrument (Magnettech, German), the EPR settings were described as follows: 20 mW microwave power, 60 s sweep time, 0.2 modulation, and 332~346 mT magnetic field. After irradiation, the EPR spectra were recorded and further analyzed by EPR software.

**AQY measurement：**

The apparent quantum yield (AQY) was determined under single wavelength Xe lamp light irradiation (λ = 313 nm), and the light intensity was measured by CEL-NP 2000 with a photodiode sensor. The AQY was calculated using the following equation:

Where, Na is Avogadro’s constant (6.022 × 10^23^ mol^-1^), h is the Planck constant (6.626 × 10^-34^ J·s), c is the speed of light (3 × 10^8^ m·s^-1^), S is the irradiation area (m^2^), *I* is the intensity of irradiation light (W·m^-2^), t is the photoreaction time (s), *λ* is the wavelength of the monochromatic light (m).

**Ultrafast transient absorption (****fs-TAS) spectroscopy:**

The femtosecond transient absorption spectroscopy (fs-TAS) measurements were performed on a Helios pump–probe system (Helios Fire, Ultrafast Systems) under ambient conditions. The pump pulses (center wavelength at 300 nm for this study; pulse energy ~1.6 μJ at the sample cell) were delivered by an optical parametric amplifier (OPerA SOLO, Coherent Inc.), which was excited by a Ti: sapphire regenerative amplifier (Astrella, 800 nm, 90 fs, 7 mJ per pulse, and 1 kHz repetition rate, Coherent Inc.). The white-light continuum probe pulses (320~650 nm) were generated by focusing a tiny portion of the 800 nm beam onto a sapphire plate. The instrument response function was determined to be ~120 fs by a routine cross-correlation procedure. The temporal and spectral profiles (chirp-corrected) of the pump-induced absorbance change of the samples were registered using an optical fiber-coupled multichannel spectrometer and further processed by a Surface Xplorer software. The sample were contained in a 0.8-mL quartz cuvette under a continuous magnetic stirring condition ensuring that the photoexcited volume of the samples was kept fresh during the fs-TA measurements.

**Computational Analysis:**

The formation of toluene clusters has broadened the UV-visible absorption peaks, allowing light from 300-370nm to effectively excite the toluene. However, the clusters in pure toluene are different in size and shape, based on the current limited calculation conditions, we choose single molecule toluene for the following calculation.

The B3LYP-D3/6-311++G(d,p) bas and the SMD solvent model was used. The toluene electronic structure before and after excitation were optimized. The electronic properties of toluene, vertical excitation energy, molecular electrostatic potential (ESP) and the evaluation of ESP are studied. All calculations were performed using the Gaussian software package. The calculation results were visualized using the GaussView package. The electrostatic potential involved in the analyses was evaluated by Multiwfn based on the highly effective algorithm proposed in Ref[^35^](#_ENREF_35).

**Formic acid measurement:**

The method for measuring formate ions by ion chromatography includes the following steps: First, mix the post-reaction organic liquid with a 100 mmol/L alkaline aqueous solution and stir thoroughly, then separate the organic and aqueous phases, and dilute the aqueous phase by a factor of 5 for ion chromatography detection. Use a D160+ ion chromatograph, equipped with systems for fluid delivery, sampling, separation, detection, and data analysis. The mobile phase is a 600 ppm NaOH solution, pumped into the system at a flow rate of 1.0 ml/min. After manual injection of the sample, separation and identification are based on the differential adsorption of ions to the stationary phase. Quantitative detection of formate ions is accomplished by measuring their conductivity.

**Description of purity of template substrate toluene:**


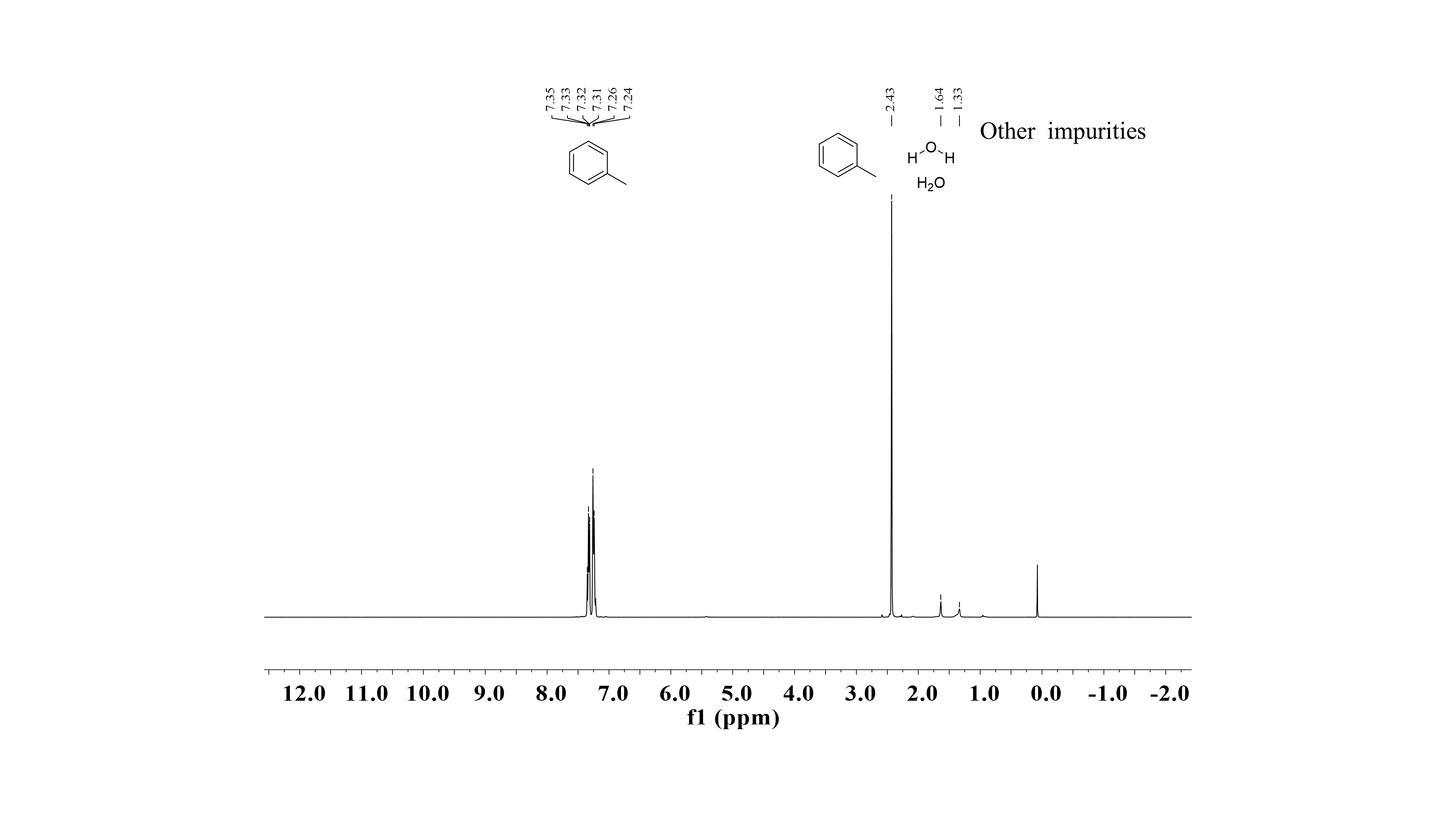


**Fig. M4a** The NMR of toluene before redistillation


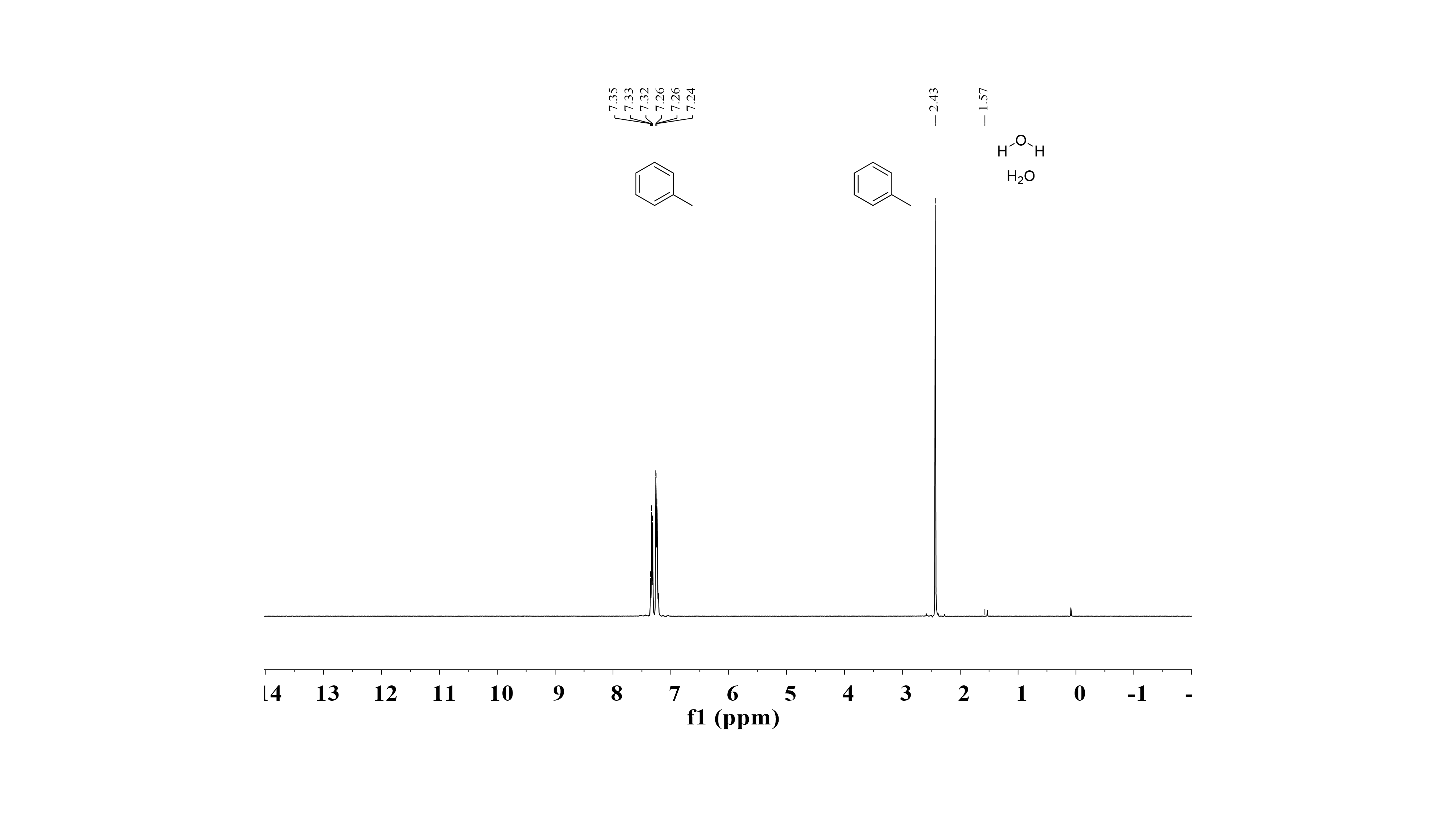


**Fig. M4b** The NMR of toluene after redistillation


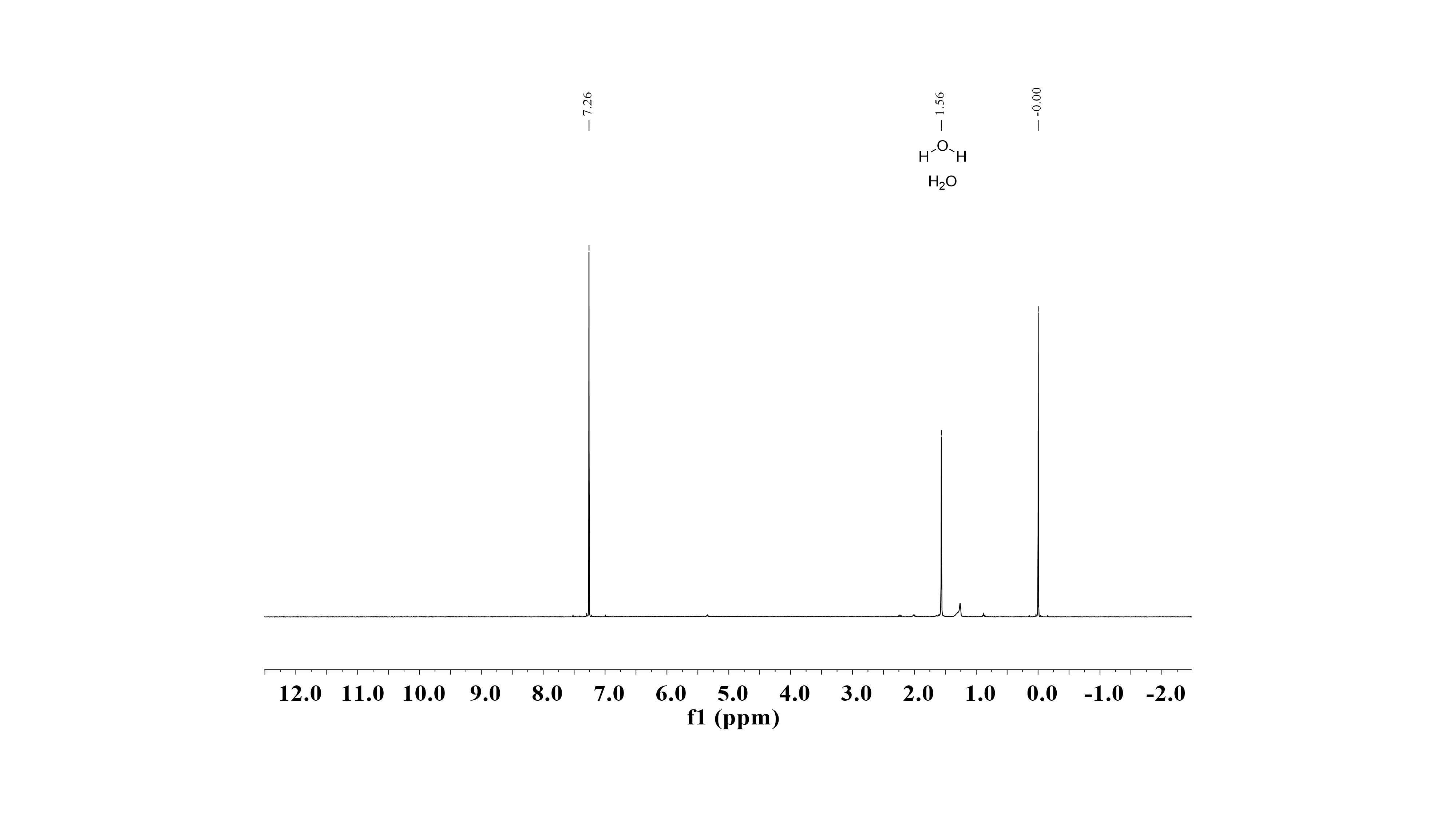


**Fig. M4c** The NMR of CDCl_3_

**Fig. M4 (a-c)** display the NMR spectra of toluene before (M4a) and after (M4b) distillation, as well as the deuterated solvent CDCl_3_ (M4c). This demonstrates that the distillation process can enhance the purity of the reaction reagent, thereby improving the accuracy of experimental data.

**The full spectrum of xenon lamp:**

**Supplementary Fig. M5** The full spectrum of xenon lamp.

The above figure, **Fig. M5**, exhibits the full spectrum of a xenon lamp used in our experiments. It can be found that no any light with wavelength less than 300 nm is provided. The main spectrum region is between 300 nm and 980 nm, and mainly concentrates between 350 nm and 760 nm, the visible light region. Only a little UVA light from 310 nm to 400 nm, is supplied by this xenon lamp.

# Figure S1


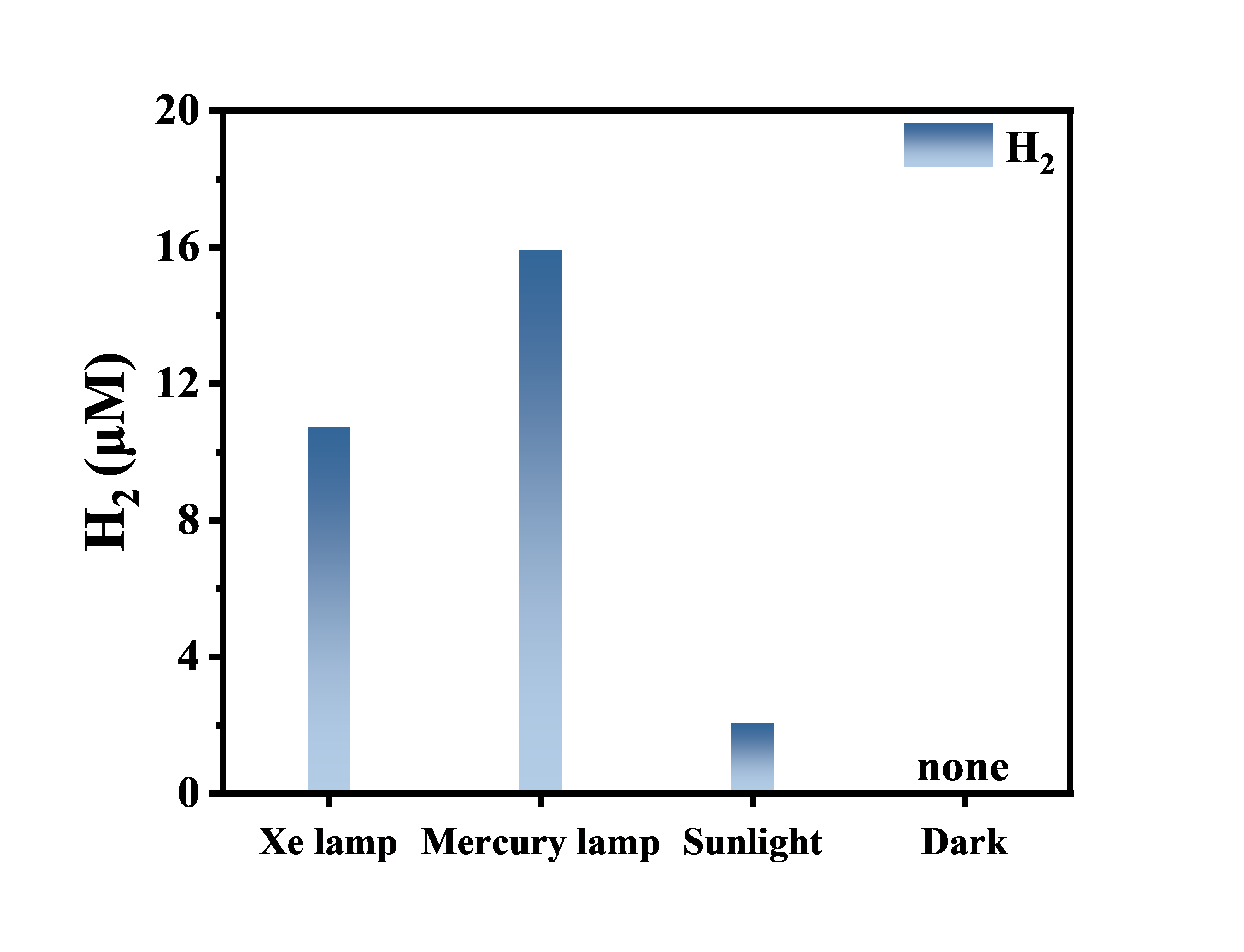


**Supplementary Fig. S1** Toluene Photosynthesize H_2_ (5 h) (natural sunlight irradiation on November 20, 2023).

As shown in **Fig. S1**, after toluene was irradiated by Xe lamp (λ = 300–1000 nm), Mercury lamp (λ = 254–365 nm), and natural sunlight for 5 h, H_2_ was generated in the reaction system with yields of 10.71, 15.92, and 2.04 μM, respectively, and no H_2_ was generated under dark conditions, even at 60 °C and overnight.

# Figure S2

**
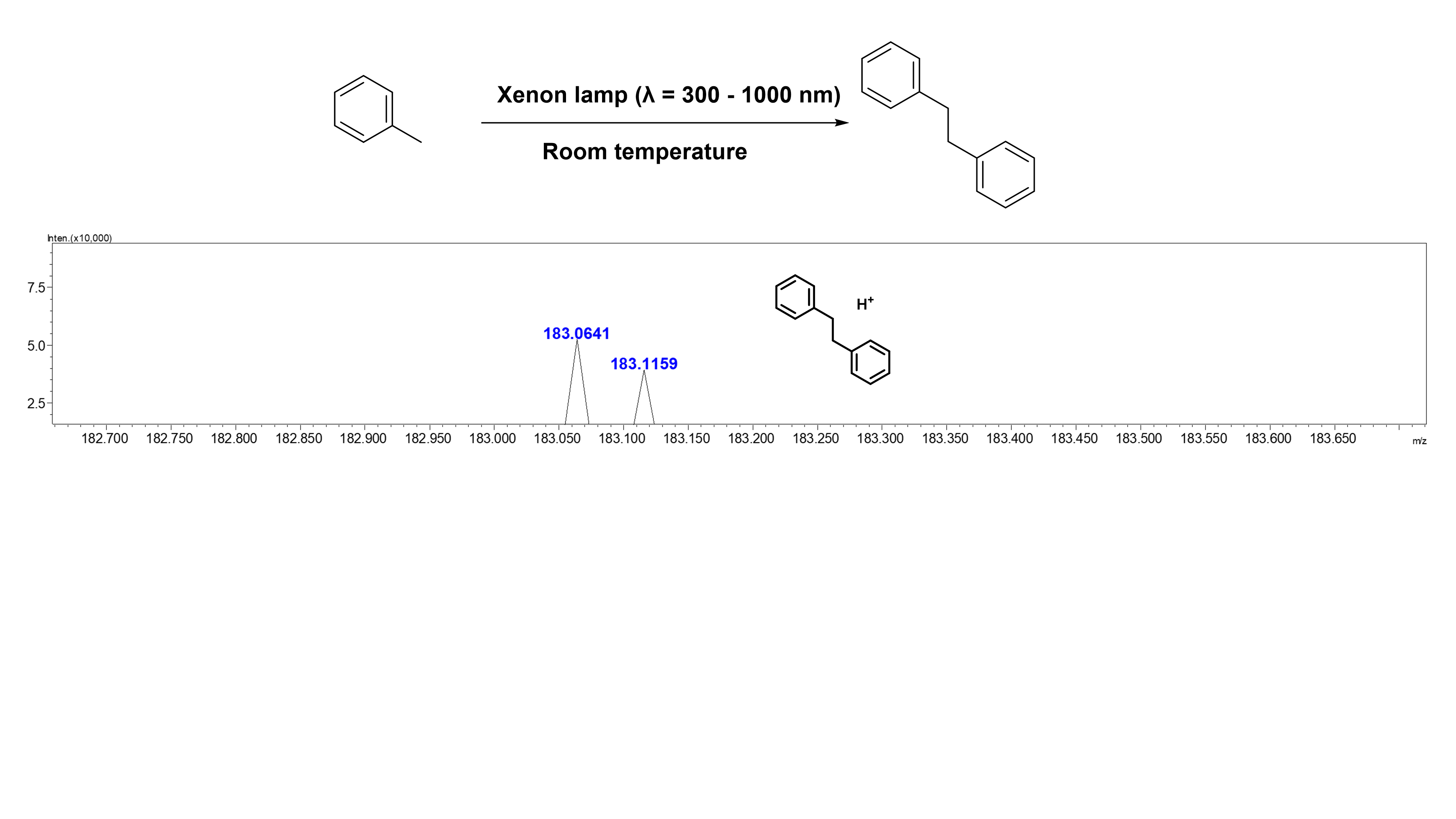
**

**Supplementary Fig. S2** The cross-coupling reaction in toluene.

As shown in **Fig. S2**, 1,2-diphenylethane was detected in the organic phase of toluene after irradiation with a xenon lamp for 5 h (**HRMS**; *m/z* (ESI) calcd for [C_14_H_14_+H^+^]^+^ = 183.1168, found =183.1159). This indicates that toluene may undergo direct homolysis of the benzylic C–H group upon photoexcitation to produce **H•** and **Bn•**, then two **Bn•** can couple to produce 1,2-diphenylethane.

# Figure S3


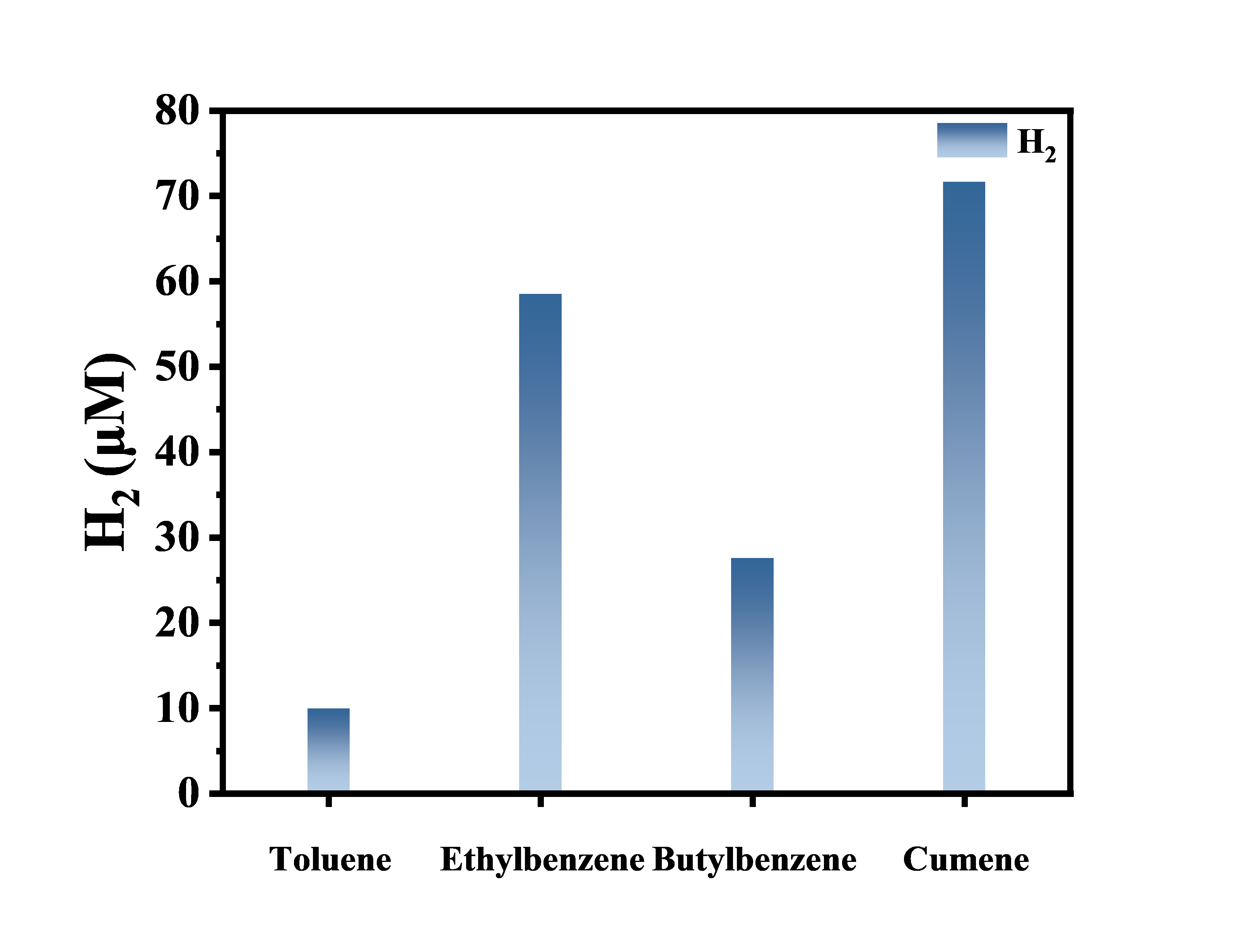


**Supplementary Fig. S3a** Benzene alkanes photosynthesize H_2_ (5 h).

As shown in **Fig. S3a**, toluene, ethylbenzene (**Fig. S3b**), butylbenzene (**Fig. S3c**) and cumene (**Fig. S3d**) were irradiated by Xe lamp (λ = 300–1000 nm) for 5 h, H_2_ was generated with yields of 10.71, 15.92, and 2.04 μM, respectively. This suggests that direct homolysis benzylic C–H is universal, and an in-depth study of this mechanism is necessary.


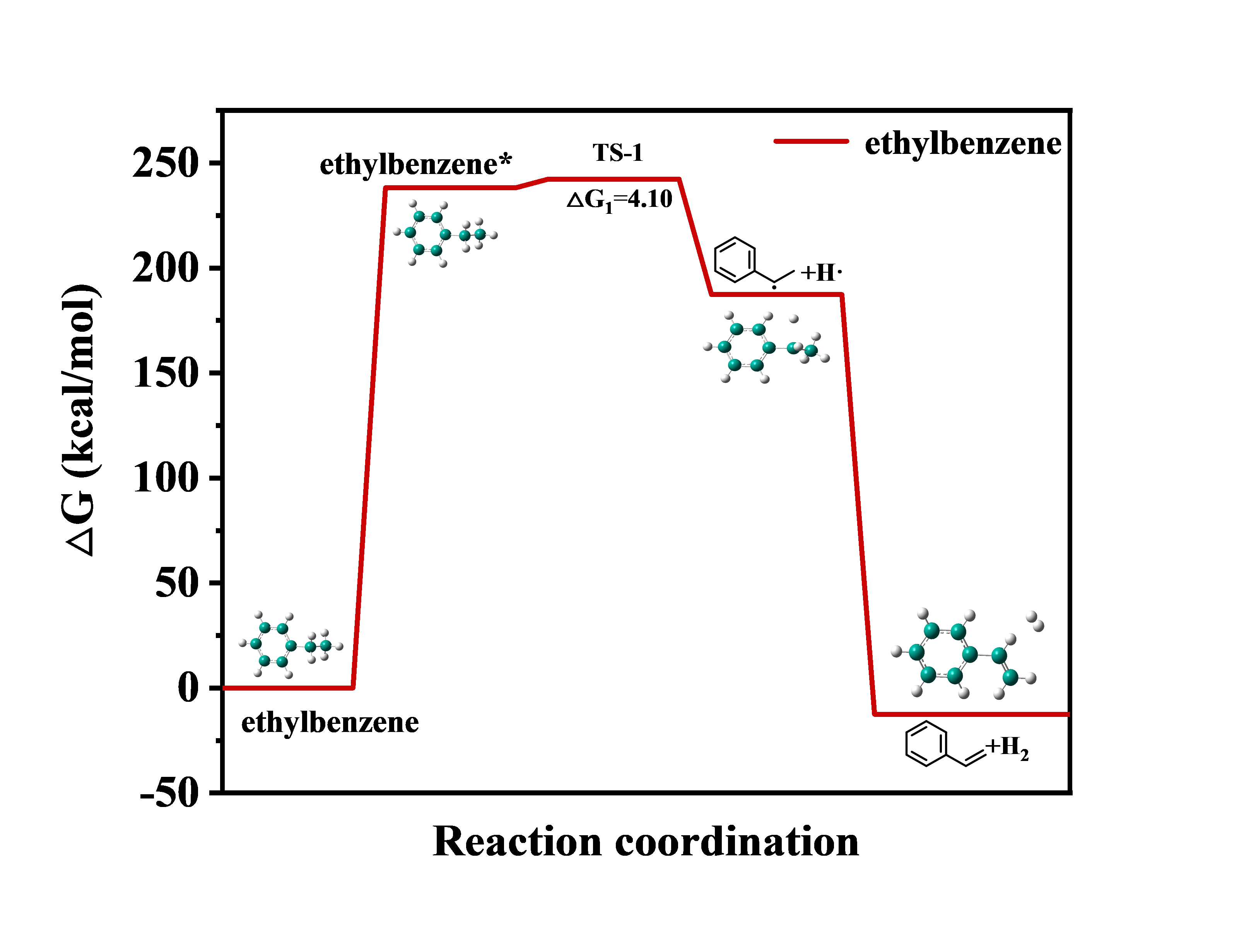


**Supplementary Fig. S3b** the hydrogen production reaction pathways of ethylbenzene.


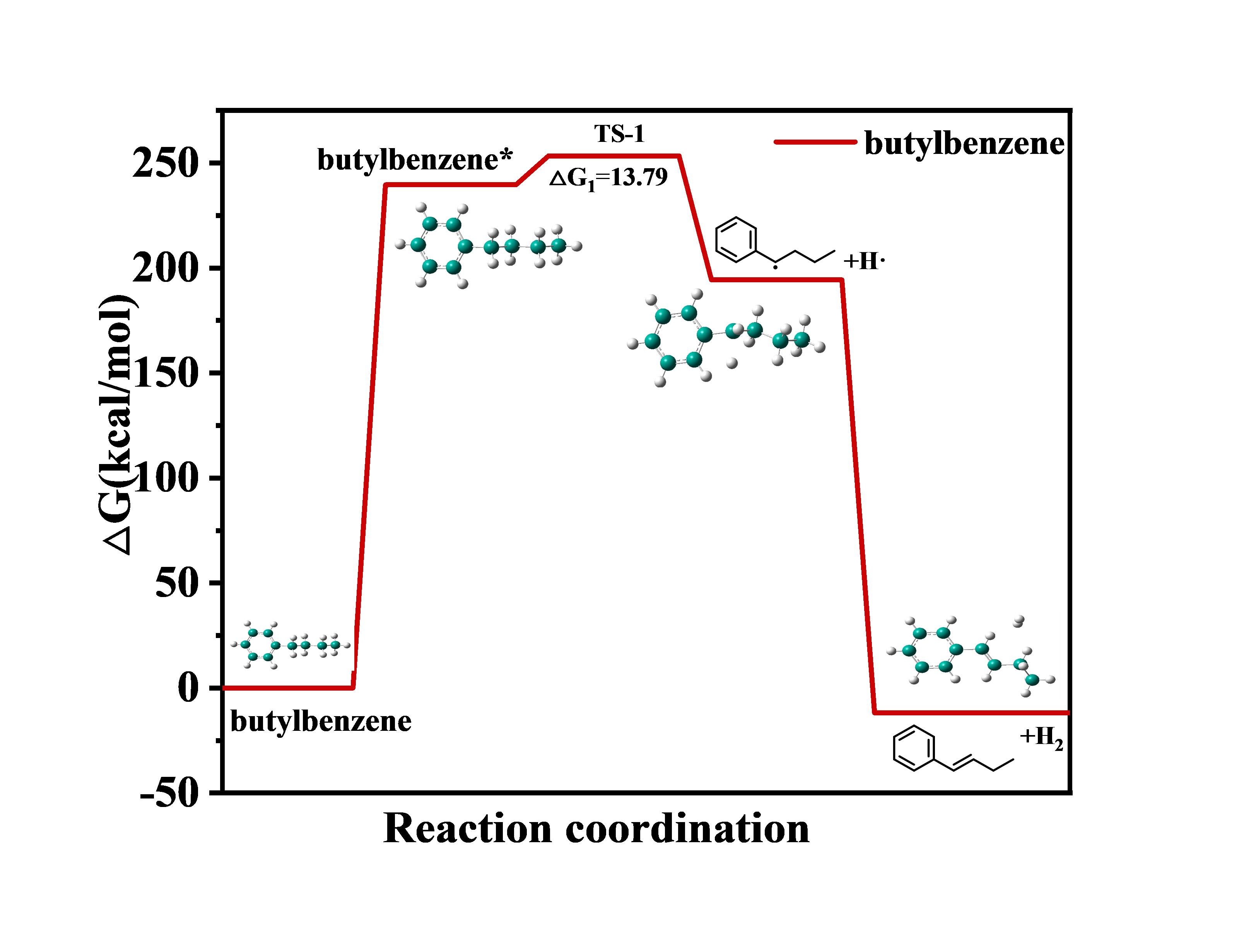


**Supplementary Fig. S3c** the hydrogen production reaction pathways of butylbenzene.


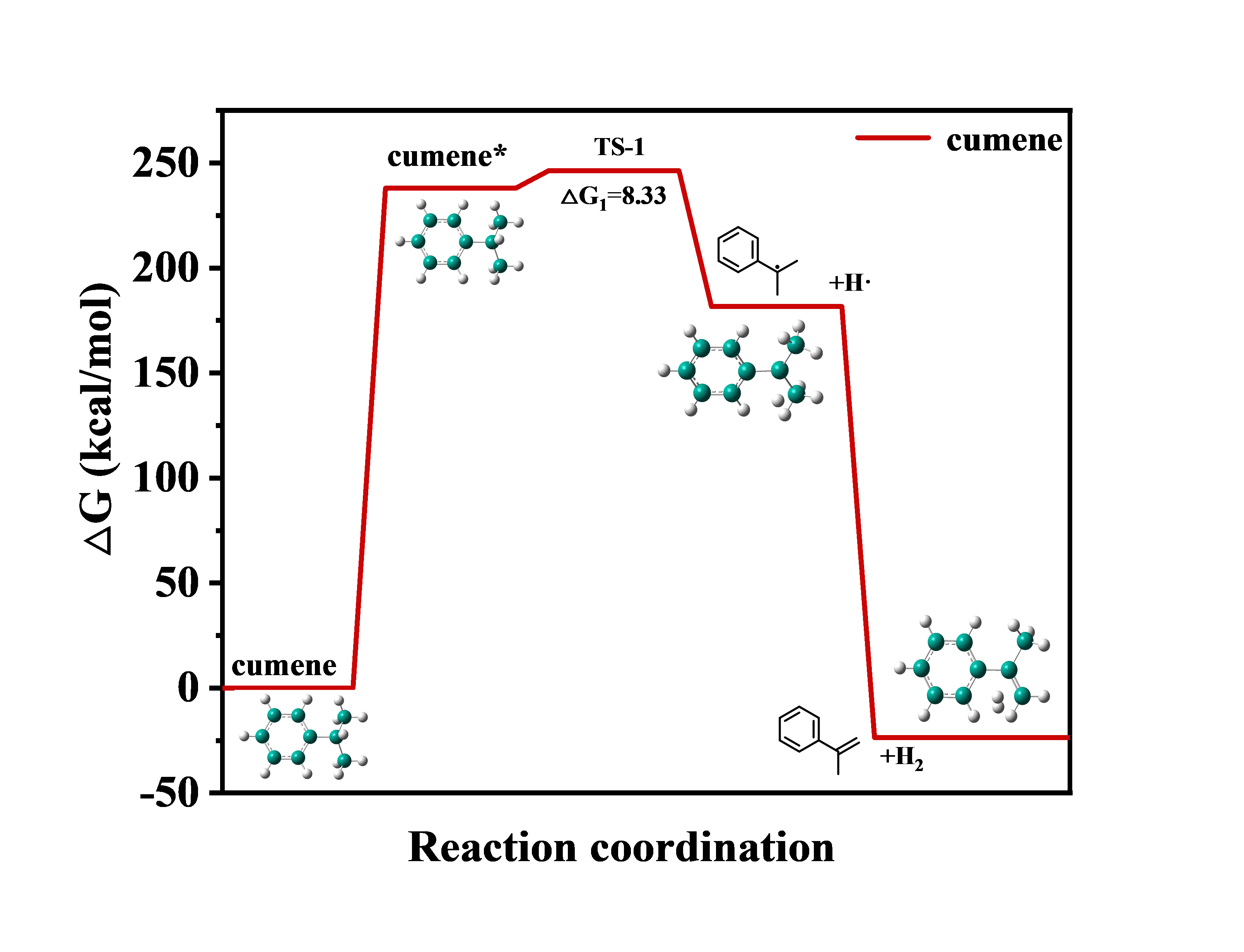


**Supplementary Fig. S3d** the hydrogen production reaction pathways of cumene.

# Figure S4

**
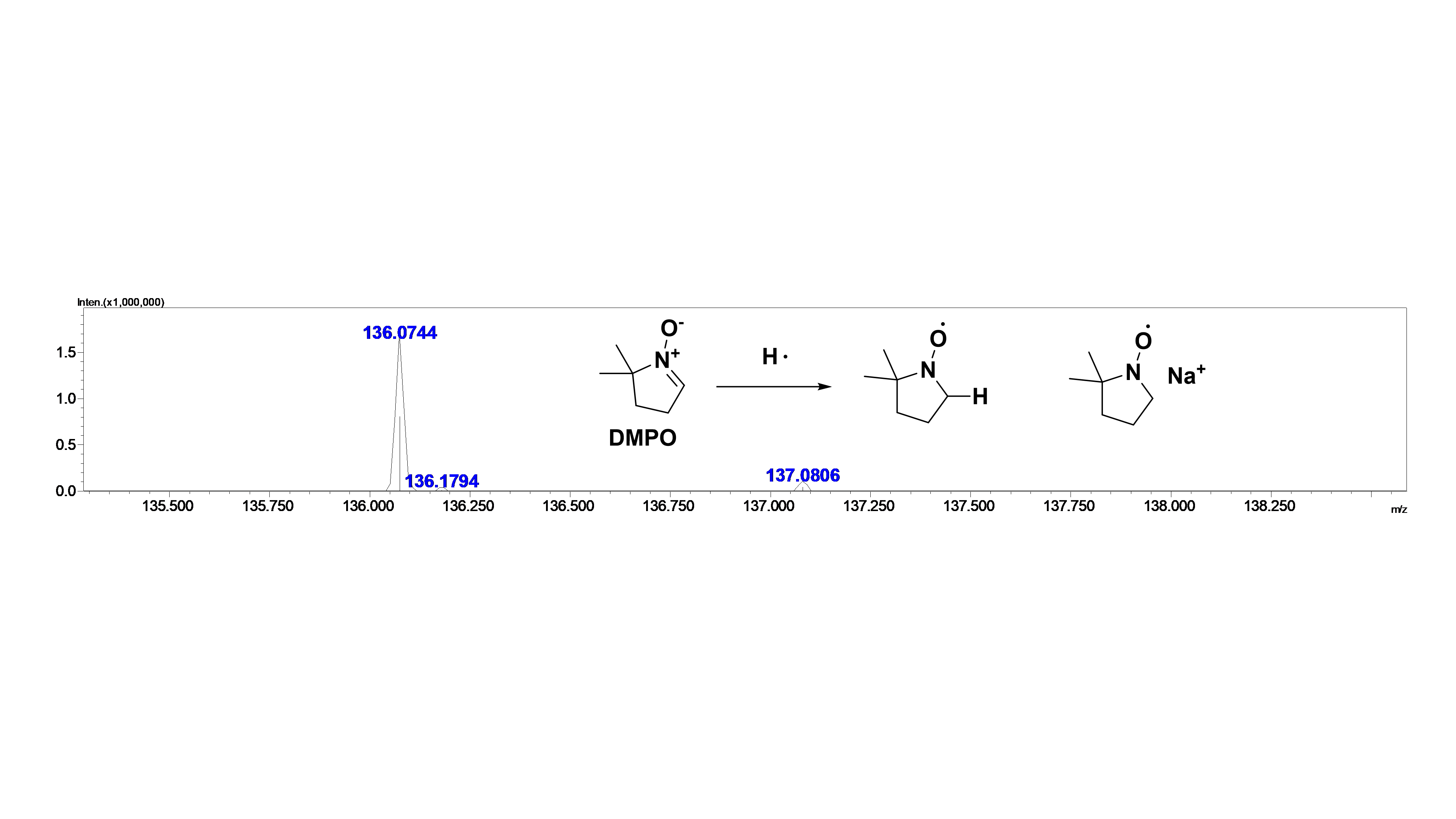
**

**Supplementary Fig. S4** The adduct of the **H•** and **DMPO**.

To gain the adduct of the **H•** and DMPO, the experiments based the condition of toluene (5 mL), mixed with DMPO (25 μL), consequent Ar (1 atm) at 20℃, irradiation of a Xe lamp (λ= 300–1000 nm) was performed. 5 minutes later, the adduct of **H•** and DMPO was detected by High-resolution mass spectra (**HRMS**; *m/z* (ESI) calcd for [C_6_H_12_NO•+Na^+^]^+^ = 137.0811, found = 137.0806) .

# Figure S5&S6

**
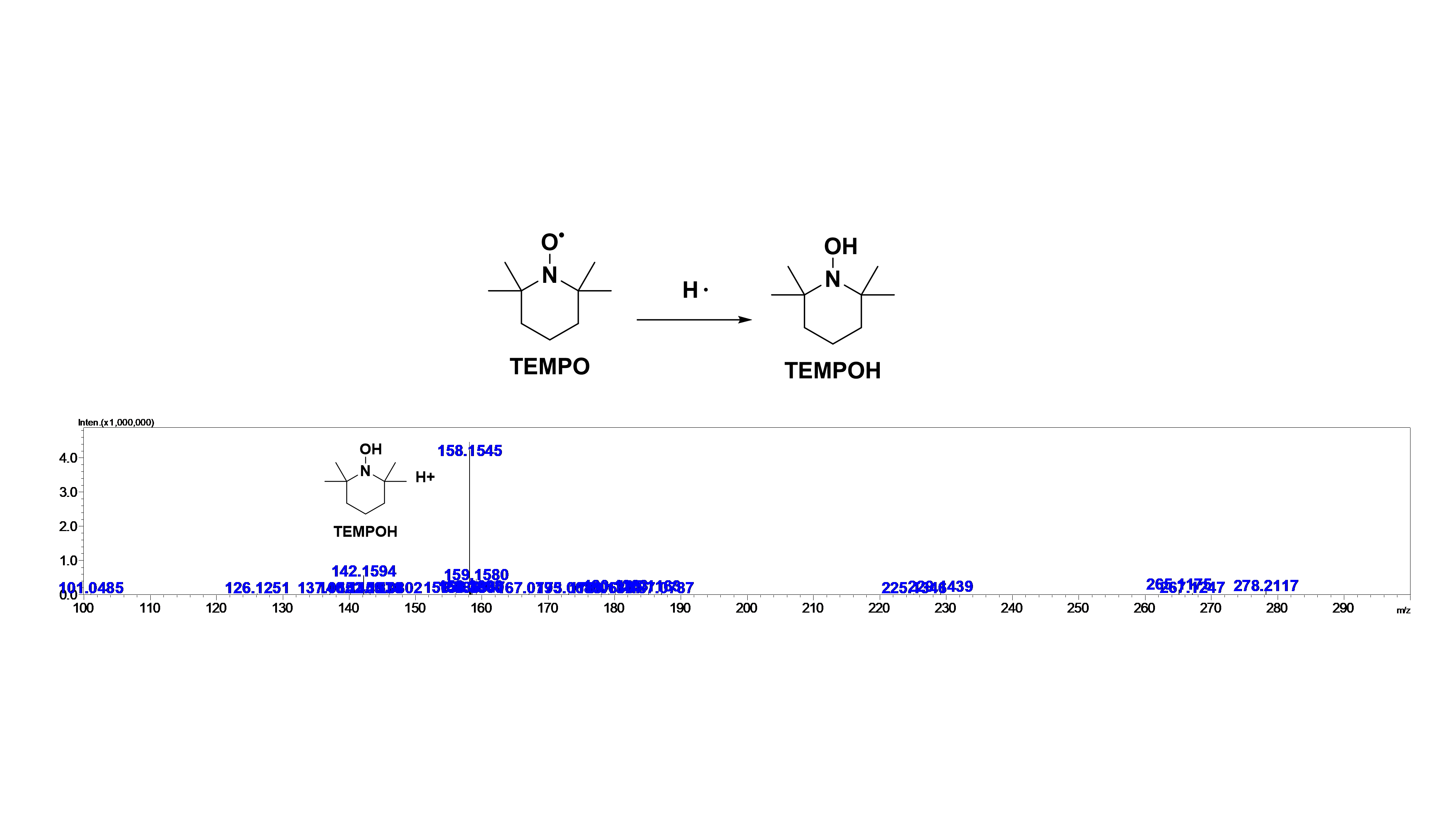
Supplementary Fig. S5** The adducts of the **H•** and TEMPO.

**
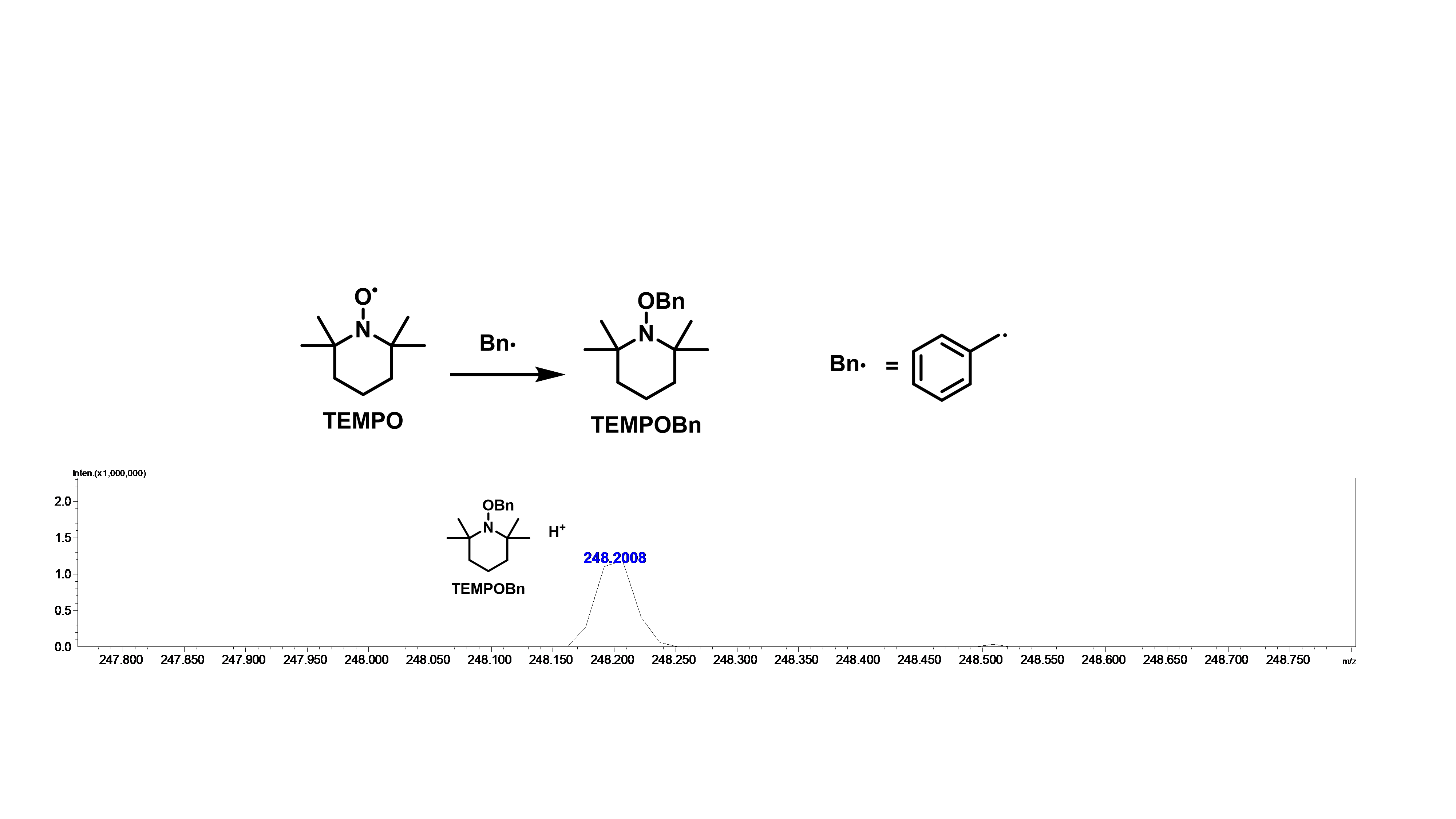
**

**Supplementary Fig. S6** The adducts of the **Bn•** and **TEMPO**.

To gain the adduct of the H• and TEMPO, the experiments based the condition of toluene (5 mL), mixed with TEMPO (25 mg), consequent Ar (1 atm) at 20℃, irradiation of a Xe lamp (λ= 300-1000 nm) was performed. 50 minutes later, the adduct of **H•** and TEMPO, TEMPOH, was detected by High-resolution mass spectra (**HRMS**; *m/z* (ESI) calcd for [C_9_H_19_NO+H^+^]^+^ = 158.1539, found = 158.1545) **(Figs. S5)**. Additionally, the formation of the H• and TEMPO adduct, TEMPOBn, was also detected (**HRMS**; *m/z* (ESI) calcd for [C_16_H_25_NO+H^+^]^+^ = 248.2009, found = 248.2008) **(Figs. S6)**.

# Figure S7

**Supplementary Fig. S7** Femtosecond transient spectra of ethyl acetate in nitrogen gas.

The bond dissociation energy of the carbonyl α-hydrogen of ethyl acetate is 96 kcal/mol^1^, which requires a photon of 297 nm to break the bond, and the photon energy of 300 nm is insufficient. However, femtosecond transient spectroscopy revealed that after the excitation of ethyl acetate by 300 nm photons, ethyl acetate would undergo a femtosecond level of vibrational relaxation, but unlike toluene, no new peaks were generated. This shows that when an organic substance is excited by a photon, it will undergo a vibrational relaxation process, which transfers energy to the more reactive bonds, and when the energy is higher than the bond dissociation energy, bond breaking will occur, but if the energy is lower than the bond dissociation energy, bond breaking will almost never occur **(Figs. S7)**.

# Figure S8&S9

**
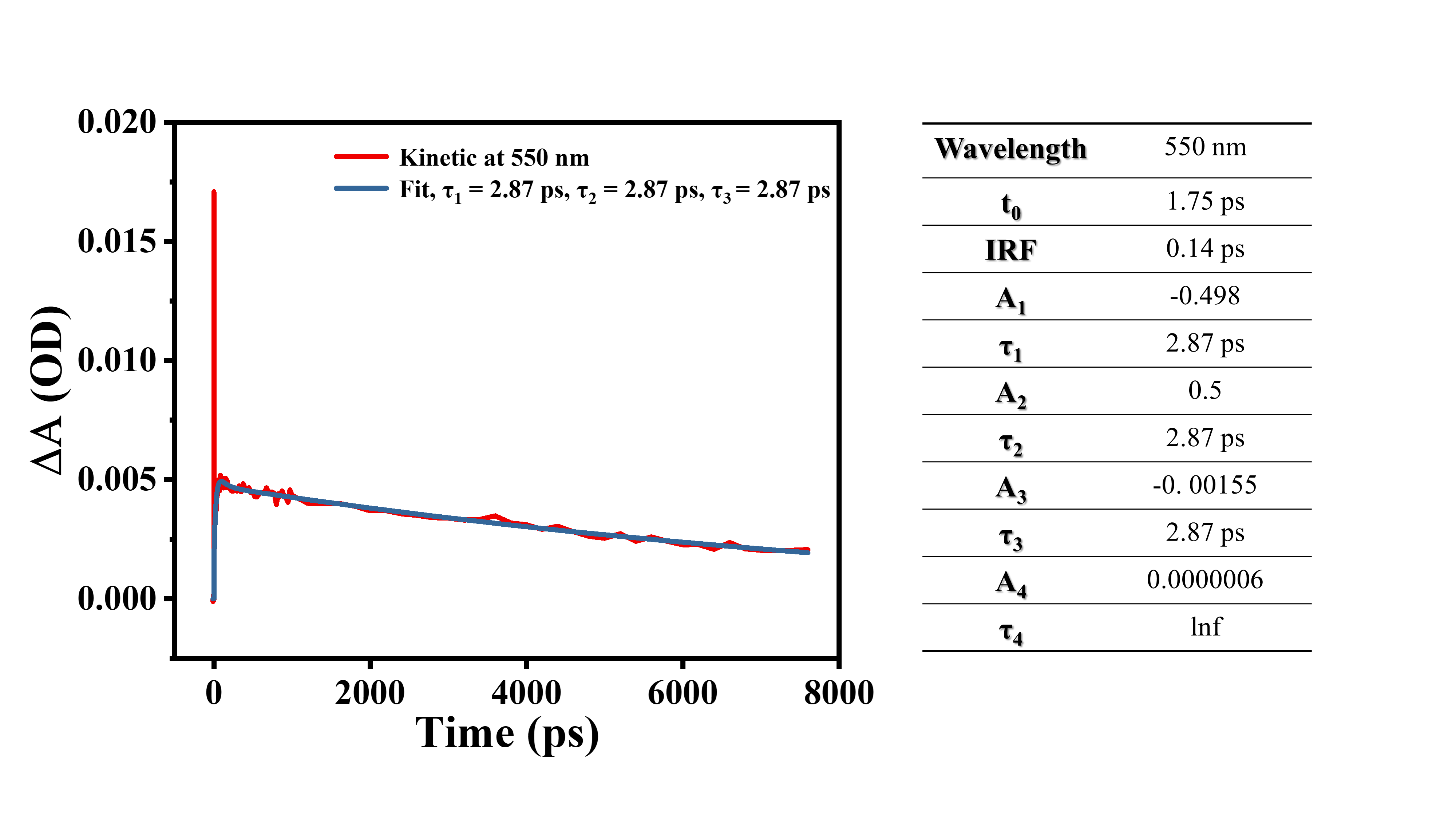
**

**Supplementary Fig. S8** The Kinetic fitting results of **II** under oxygen-free conditions at 550 nm.

**Supplementary Fig. S9** The processes of formation and recombination of **Bn•** and **H•**.

In order to investigate the kinetic of region **I** **(Fig. 2c)**, a kinetic fitting study was performed of toluene at 550 nm. The results as shown in **Fig. S8**, the region **I** revealed three kinetic processes. Two mainly reversible processes (approximately 2.87 ps) of those, which can be attributed to the formation and recombination of **Bn•** and **H•** resulting from the homolysis cleavage of the C–H bond. The third process is speculated to be the generation of 1,2-diphenylethane by **Bn•** coupling (approximately 2.87 ps).

# Figure S10

**
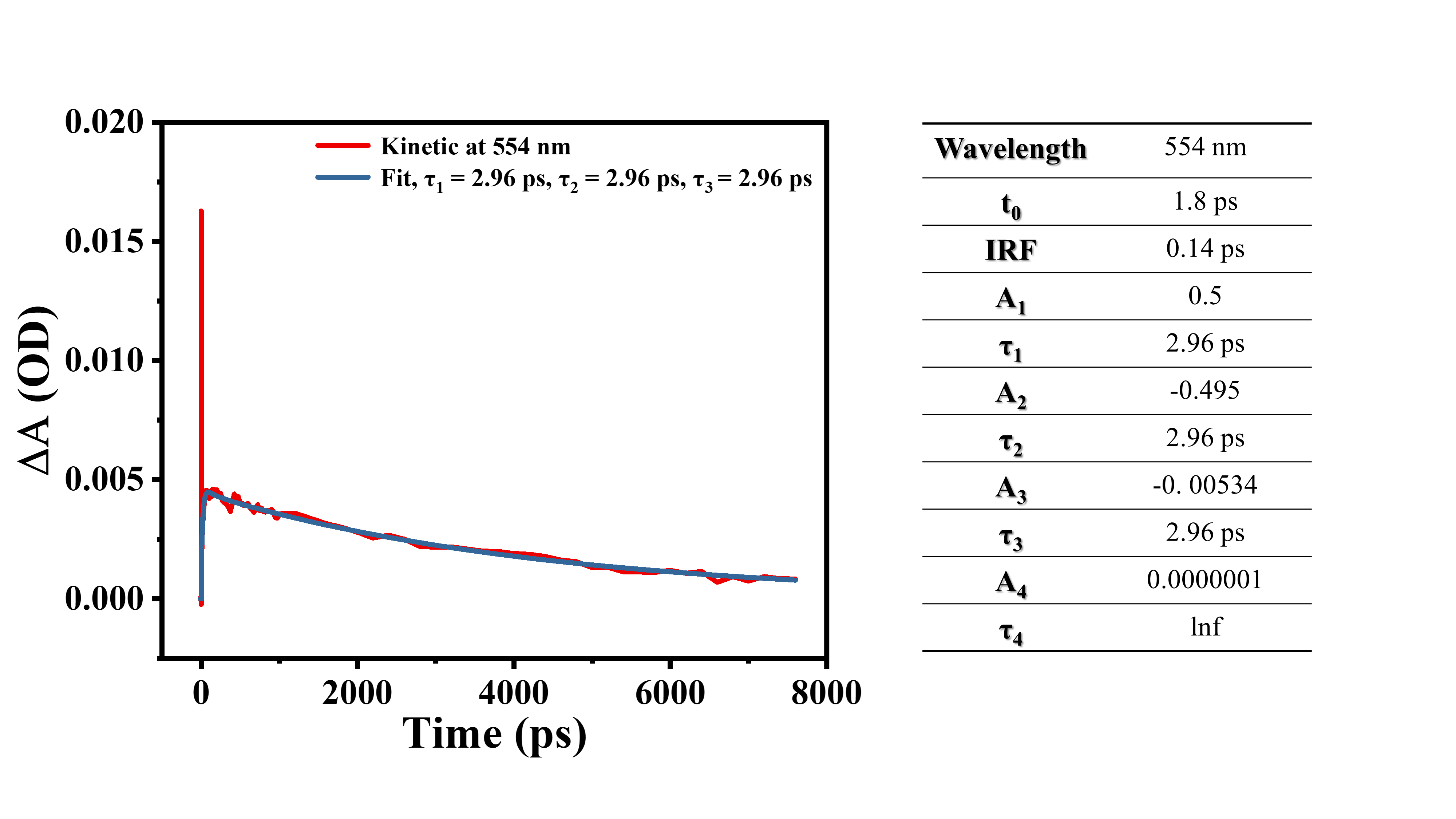
**

**Supplementary Fig. S10** The Kinetic fitting results of **II** under oxygen conditions at 564.6 nm.

In order to investigate the kinetic of toluene corresponding to the region **II (Fig. 2d)** under oxygen conditions, a kinetic fitting study was performed at a wavelength of 554 nm. The results as shown in **Fig. S10**, the region **II** exhibit three kinetic processes (**Fig. 2d & Fig. S10**), with two mainly reversible processes exhibiting equal timescales of approximately 2.96 ps. It was inferred that this corresponded to the benzylic C–H homolysis of toluene, resulting in the generation of **Bn•** and **H•**, as well as the recombination. Another process would be the formation of 1,2-diphenylethane, estimated to occur within a timescale of approximately 2.96 ps.

# Table S1

Using B3LYP-D3/6-311++G(d,p) to calculate the electronic transition energy of singlet toluene, as well as the comparison between the experimental and calculated values of the absorption wavelength, the electronic transition orbit and the transition properties, the calculation results are shown in **Table S1**. It can be observed that there is a strong absorption band at 249.15 nm for toluene, corresponding to the S1 state, where the predominant orbital transition is from the 25th to the 26th orbital. Following excitation, the HOMO molecular orbital is primarily distributed in a π-bonding manner within the benzene ring, while sigma orbitals are present in the methyl group. Additionally, the LUMO exhibits anti-bonding orbital properties, which enhances the reactivity of the C-H bond in the methyl group upon excitation, indicating a π_H-1_→π_L_^*^ transition.

**Table S1**. Calculation conditions of B3LYP-D3/6-311++G(d,p) and experimentally obtained electron orbital transition energies and absorption wavelengths of toluene

| States | Character | Singlet energies(nm) | | transition energies(eV) | Orbitals |
| --- | --- | --- | --- | --- | --- |
|  |  | Expt. |  | Calc. |  |
| S1 | π_H-1_→π_L_^*^ | 249.15 |  | 4.976 | 25→26 |


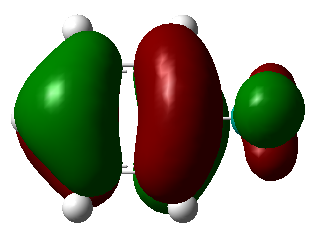

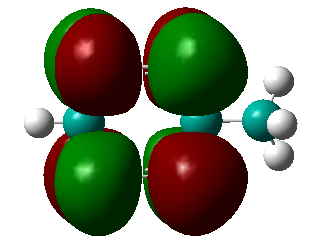


| 25: π_H-1_ | 26: π_L_^*^ |
| --- | --- |

# Figure S11

**
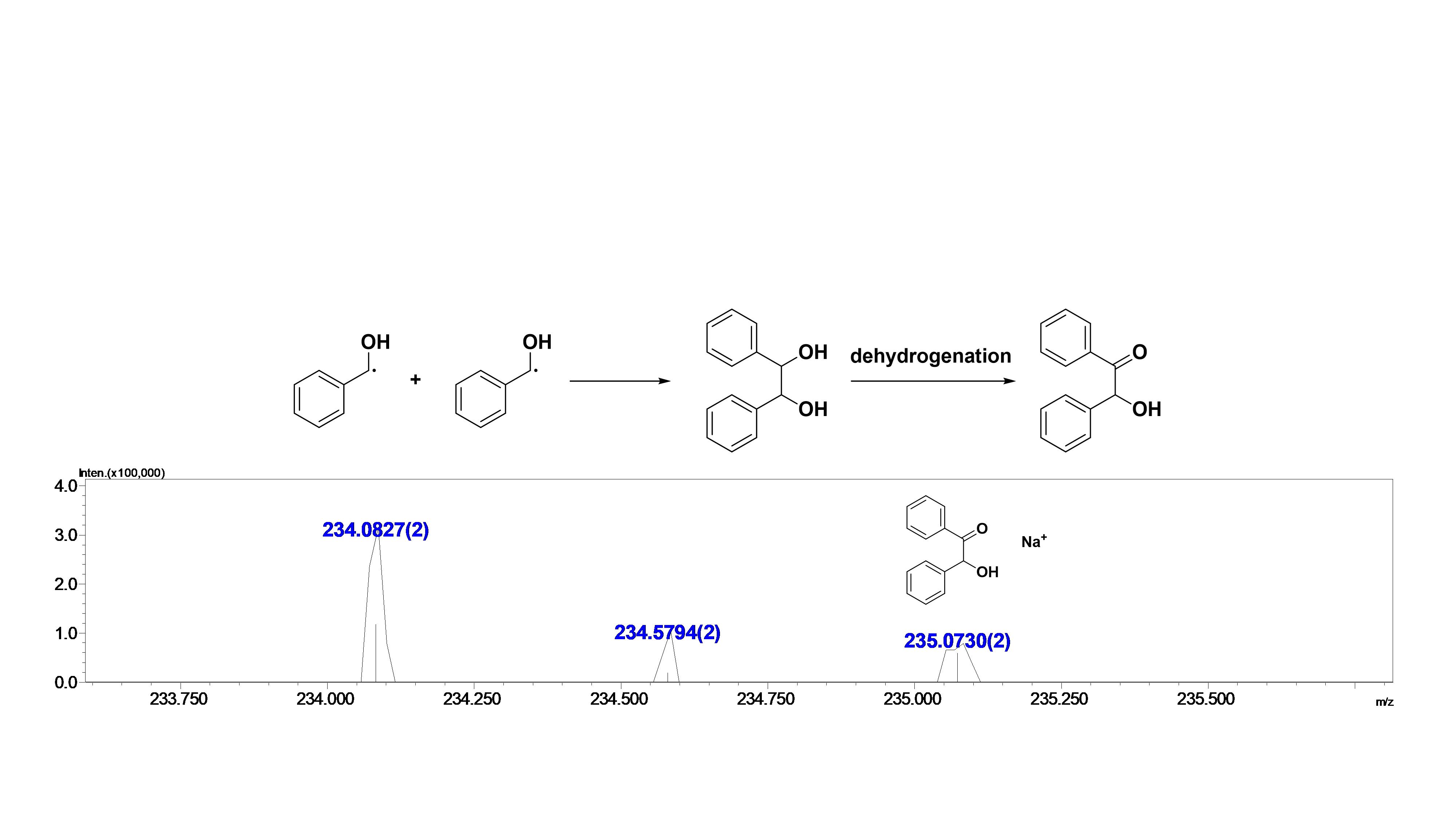
**

**Supplementary Fig. S11** The formation of benzoin.

In order to obtain the possible products in the organic phase after benzyl alcohol photoexcitation, the experimental conditions were benzyl alcohol (5 mL), consequent N_2_ (1 atm) at 20℃, irradiation of a Xe lamp (λ= 300–1000 nm) was performed. 5 hours later, the benzoin was detected by high-resolution mass spectrometry (**HRMS**; m/z (ESI) calcd for [C_14_H_12_O_2_+Na^+^]^+^ = 235.0730, found = 235.0730).

# Figure S12

**
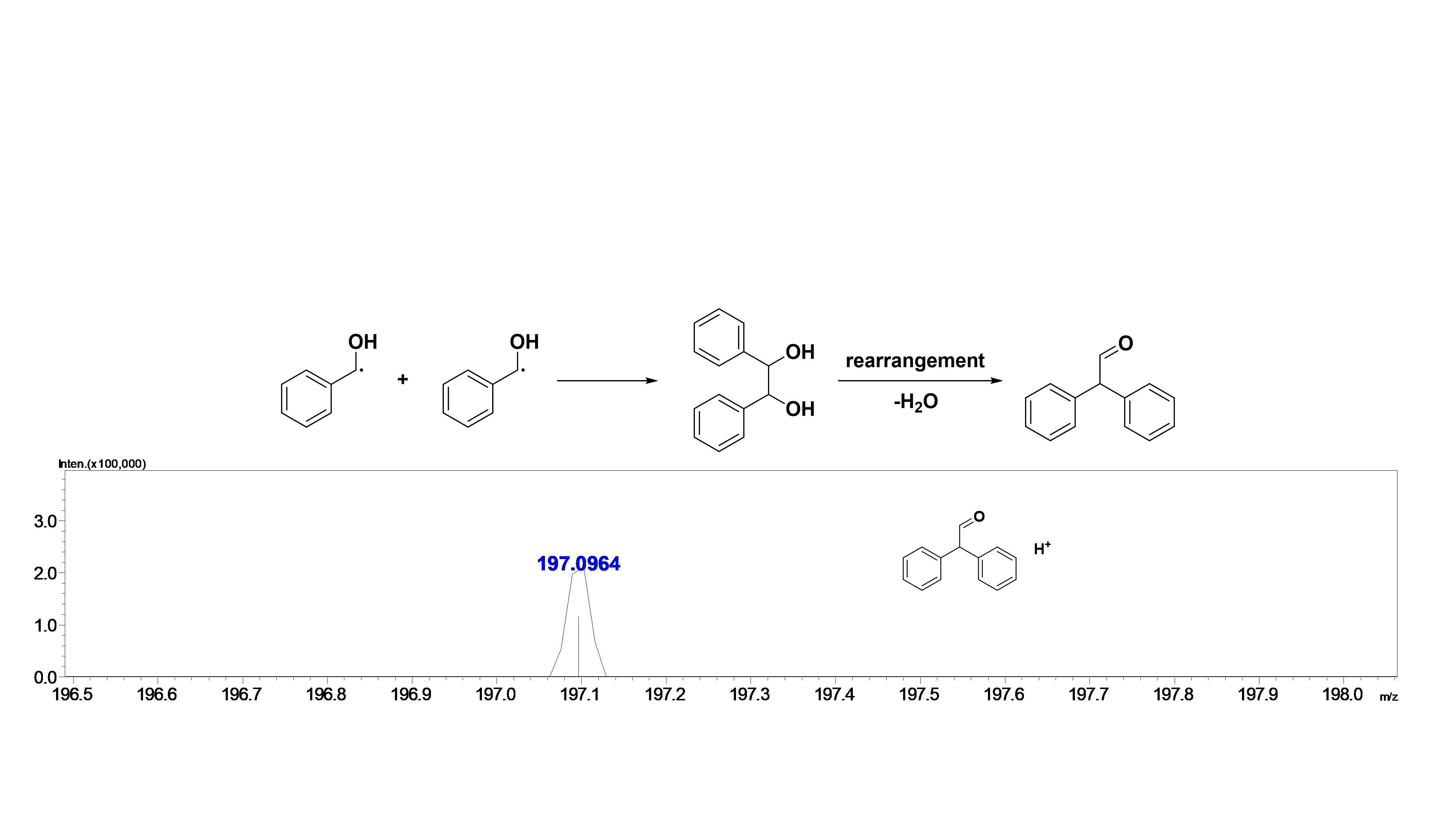
**

**Supplementary Fig. S12** The formation of diphenylacetaldehyde.

In order to obtain the possible products in the organic phase after benzyl alcohol photoexcitation, the experimental conditions were benzyl alcohol (5 mL), consequent N_2_ (1 atm) at 20℃, irradiation of a Xe lamp (λ= 300–1000 nm) was performed. 5 hours later, the diphenylacetaldehyde was detected by high-resolution mass spectrometry (**HRMS**; m/z (ESI) calcd for [C_14_H_12_O+H^+^]^+^ = 197.0961, found = 197.0964).

# Figure S13

**
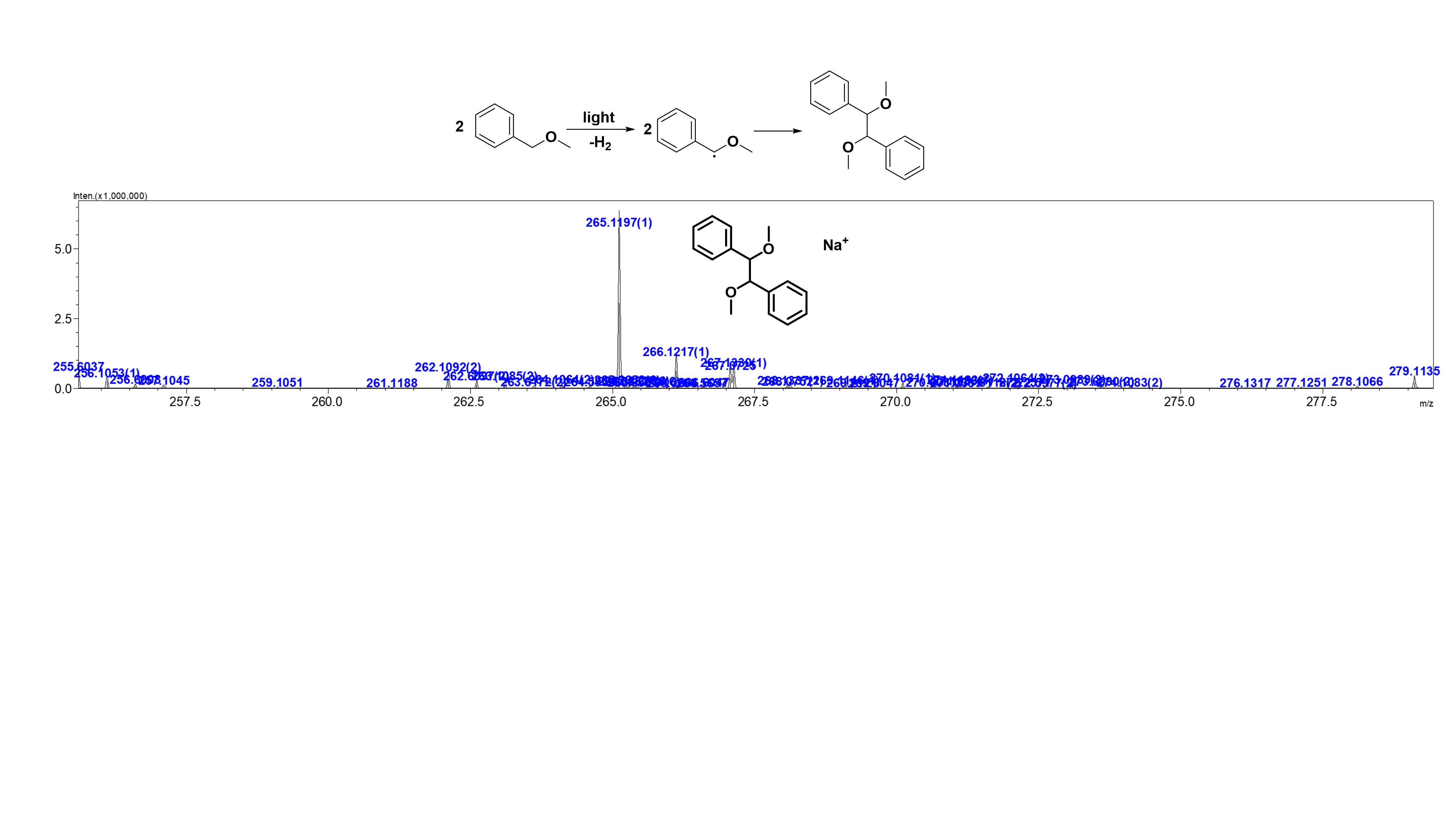
**

**Supplementary Fig. S13** The formation of 1,2-dimethoxy-1,2-diphenylethane.

As shown in **Fig. S13**, the benzylic C–H of benzyl methyl ether homolysis undergoes homolysis to form **H•** and benzyl radical, the benzyl radical would couple with another benzyl radical to generate 1,2-dimethoxy-1,2-diphenylethane**,** which was detected by high-resolution mass spectra (**HRMS**; *m/z* (ESI) calcd for [C_16_H_18_O_2_+Na^+^]^+^ = 265.1199, found = 265.1197). The experimental conditions were benzyl methyl ether (5 mL), consequent N_2_ (1 atm) at 20℃, irradiation of a Xe lamp (λ= 300–1000 nm), reaction time 5 h.

# Figure S14

**
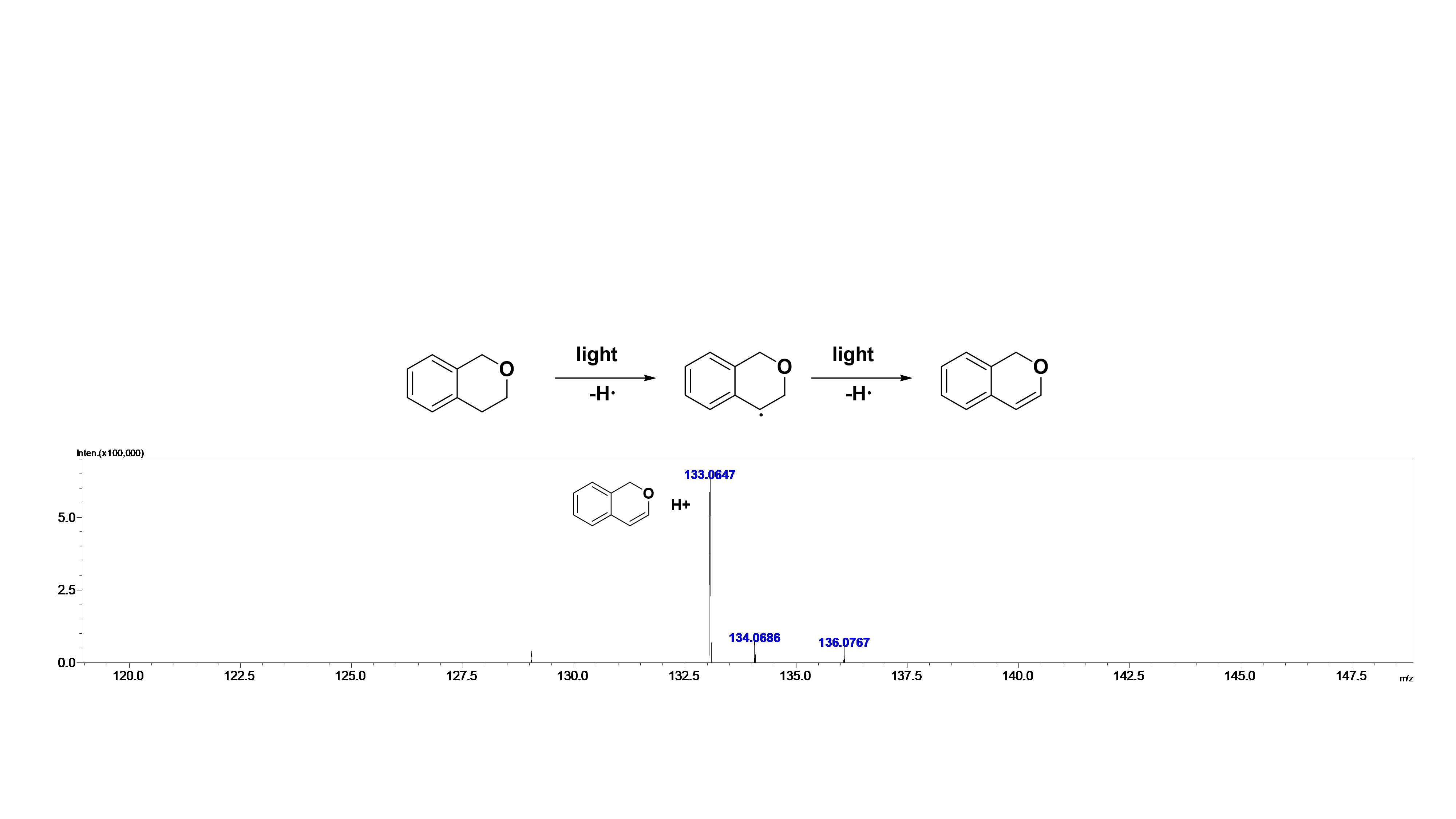
**

**Supplementary Fig. S14** The formation of 1H-2-Benzopyran.

As shown in **Fig. S14**, the benzylic C-H of isochroman undergoes homolysis to form **H•** and benzyl radical, the benzyl radical would undergo dissociation of **H•** to form 1H-2-Benzopyran**,** which was detected by high-resolution mass spectra (**HRMS**; *m/z* (ESI) calcd for [C_9_H_8_O+H^+^]^+^ = 133.0648, found = 133.0647). The experimental conditions were isochroman (5 mL), consequent N_2_ (1 atm) at 20℃, irradiation of a Xe lamp (λ= 300–1000 nm), reaction time 5 h.

# Figure S15

**
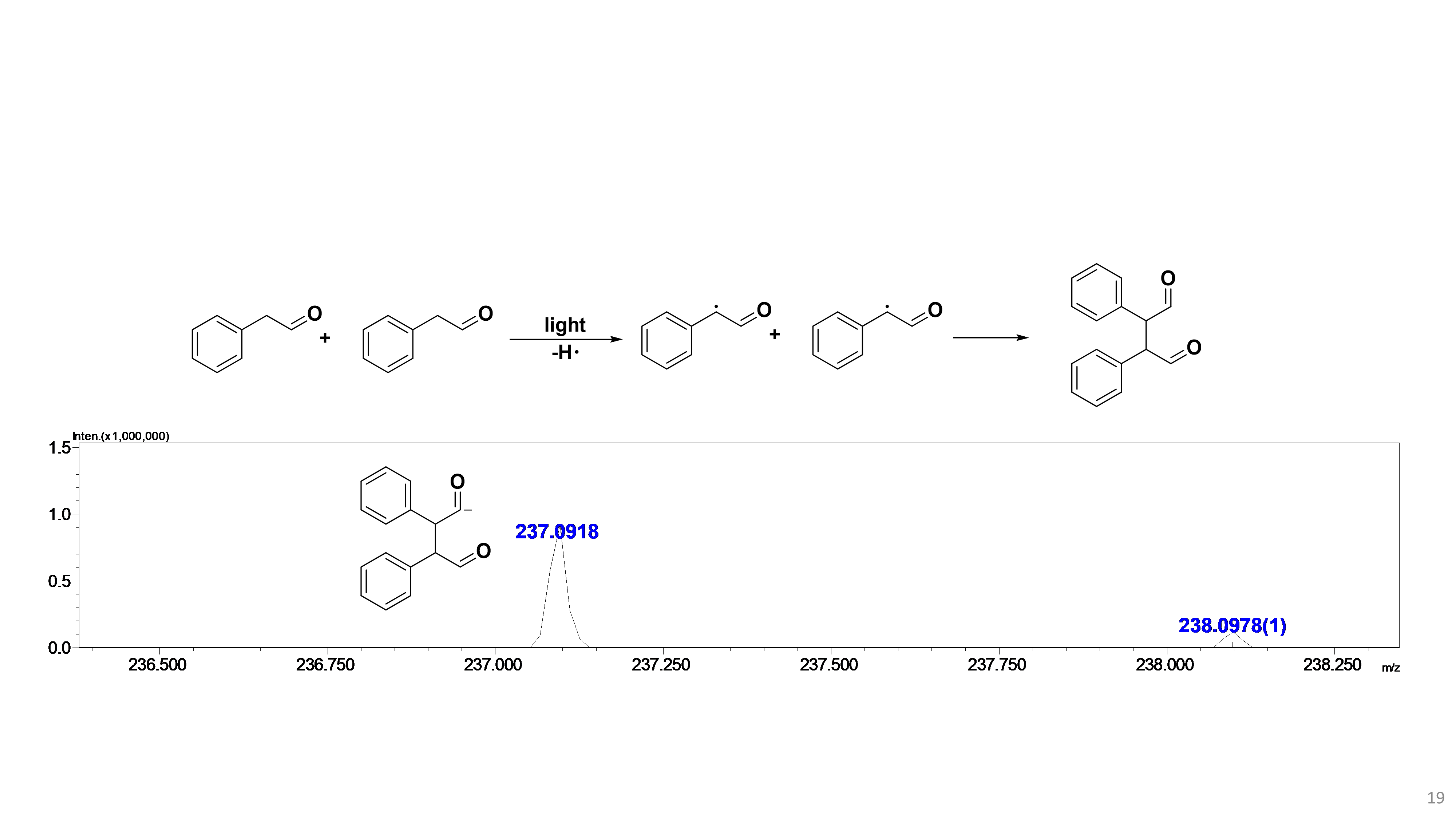
**

**Supplementary Fig. S15** The formation of 2,3-diphenylsuccinaldehyde.

As shown in **Fig. S15**, the benzylic C–H of phenylacetaldehyde undergoes homolysis to form **H•** and benzyl radical, the benzyl radical would couple with another benzyl radical to form 2,3-diphenylsuccinaldehyde**,** which was detected by high-resolution mass spectra (**HRMS**; *m/z* (ESI) calcd for [C_16_H_14_O_2_-H^+^]^-^ = 237.0921, found = 237.0918). The experimental conditions were phenylacetaldehyde (5 mL), consequent N_2_ (1 atm) at 20℃, irradiation of a Xe lamp (λ= 300–1000 nm), reaction time 5 h.

# Figure S16

**
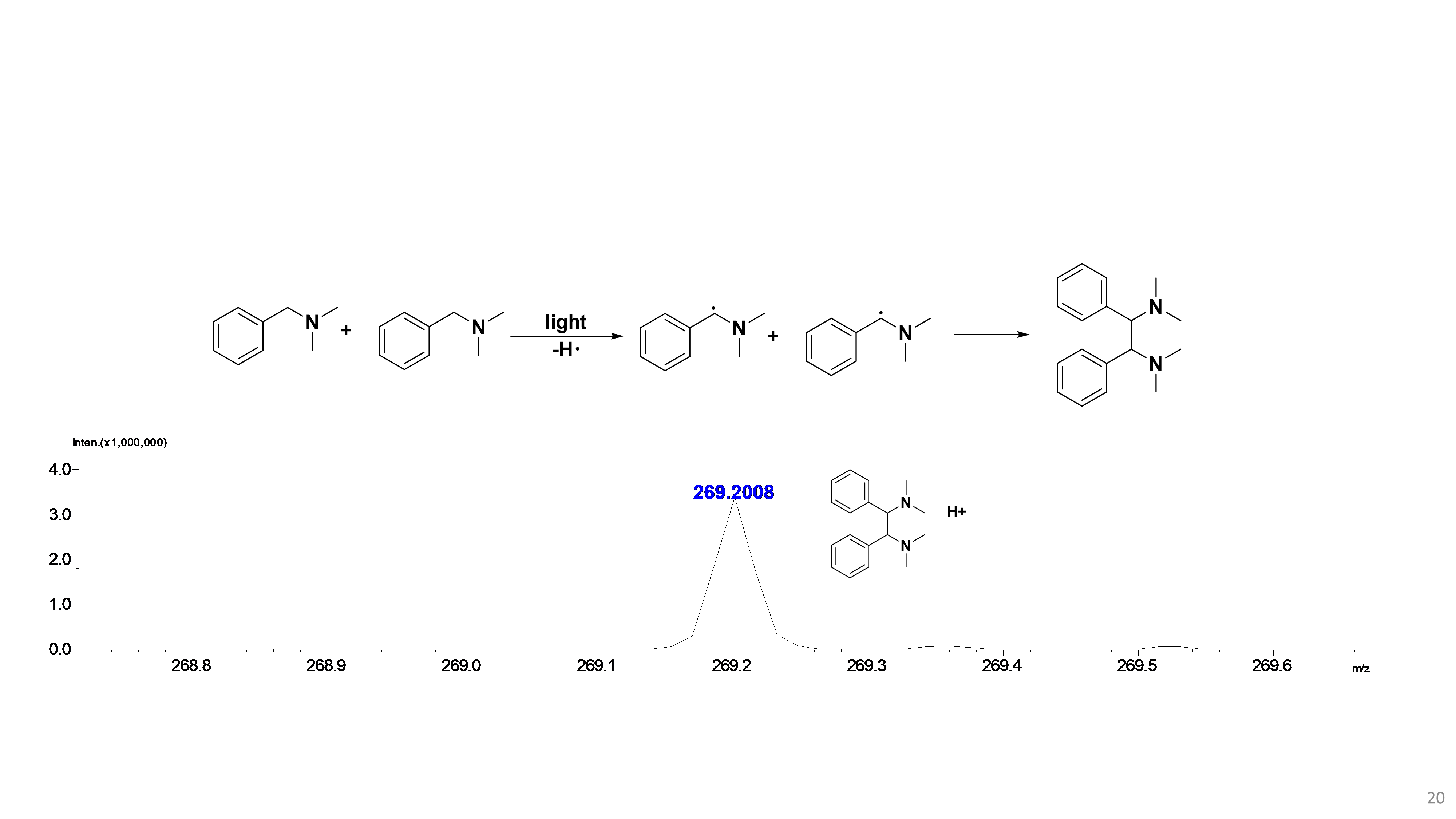
**

**Supplementary Fig. S16** The formation of N1,N1,N2,N2-tetramethyl-1,2-diphenylethane-1,2-diamine.

As shown in **Fig. S16**, the benzylic C–H of N,N-dimethylbenzylamine homolysis cleavage to produce **H•** and benzyl radical, the benzyl radical would couple with another benzyl radical to form N1,N1,N2,N2-tetramethyl-1,2-diphenylethane-1,2-diamine**,** which was detected by high-resolution mass spectra (**HRMS**; *m/z* (ESI) calcd for [C_18_H_24_N_2_+H^+^]^+^ = 269.2012, found = 269.2008). The experimental conditions were N,N-dimethylbenzylamine (5 mL), consequent N_2_ (1 atm) at 20℃, irradiation of a Xe lamp (λ= 300–1000 nm), reaction time 5 h.

# Figure S17

**
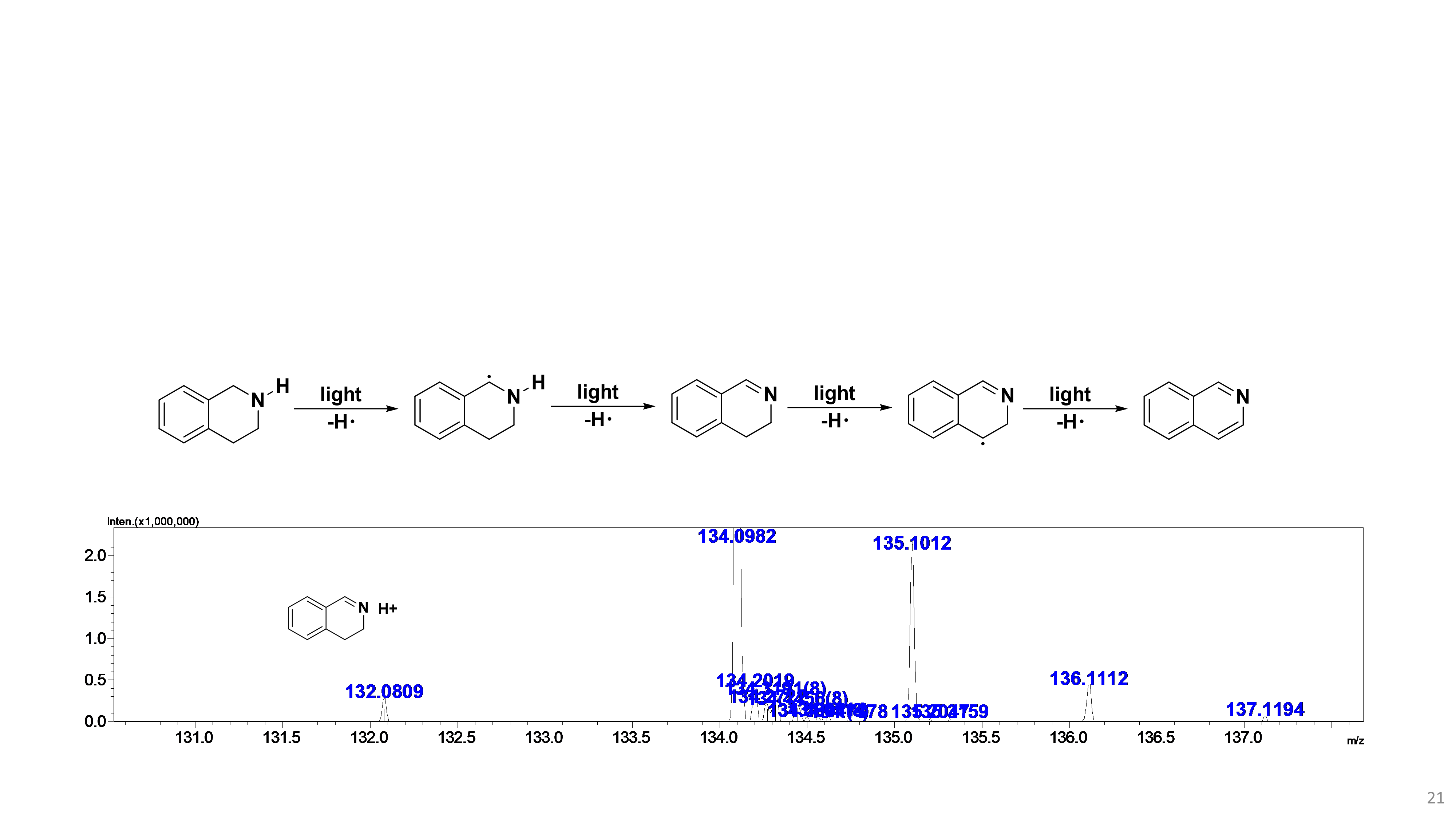
**

**Supplementary Fig. S17** The formation of 3,4-dihydroisoquinoline.

As shown in **Fig. S17**, the benzylic C–H of tetrahydroisoquinoline homolysis cleavage to produce **H•** and benzyl radical, the benzyl radical would undergo dissociation of **H•** to form 3,4-dihydroisoquinoline**,** which was detected by high-resolution mass spectra (**HRMS**; *m/z* (ESI) calcd for [C_9_H_9_N+H^+^]^+^ =132.0808, found = 132.0809). The experimental conditions were tetrahydroisoquinoline (5 mL), consequent N_2_ (1 atm) at 20℃, irradiation of a Xe lamp (λ= 300–1000 nm), reaction time 5 h.

# Figure S18

**
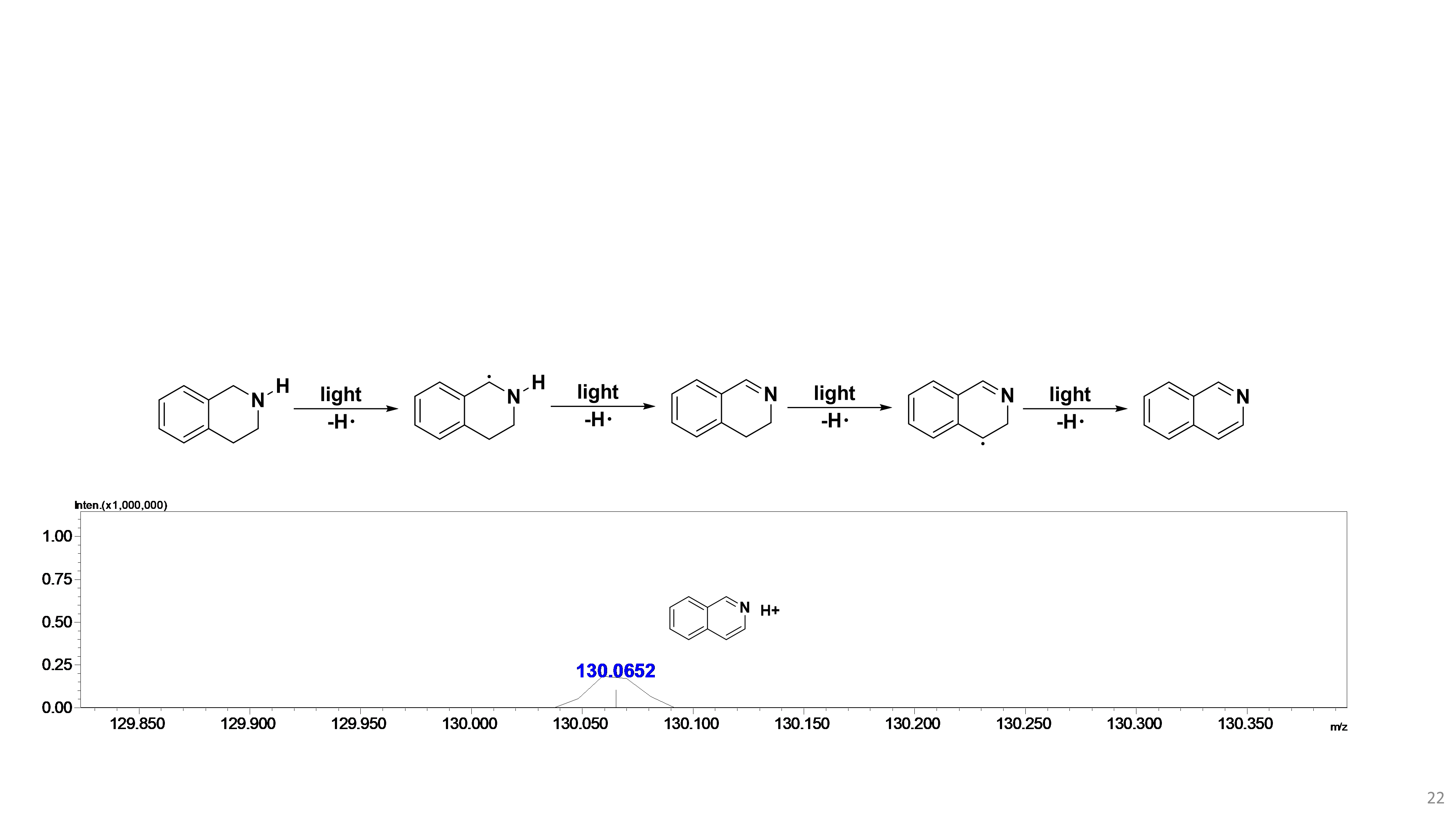
**

**Supplementary Fig. S18** The formation of isoquinoline.

As shown in **Fig. S18**, the benzylic C–H of tetrahydroisoquinoline homolysis cleavage to produce **H•** and benzyl radical, the benzyl radical would undergo dissociation of **H•** to form 3,4-dihydroisoquinoline**,** and3,4-dihydroisoquinoline undergo dissociation of **H•** to form isoquinoline, which was detected by high-resolution mass spectra (**HRMS**; *m/z* (ESI) calcd for [C_9_H_7_N+H^+^]^+^ =130.0651, found = 130.0652). The experimental conditions were tetrahydroisoquinoline (5 mL), consequent N_2_ (1 atm) at 20℃, irradiation of a Xe lamp (λ= 300–1000 nm), reaction time 5 h.

# Figure S19

**
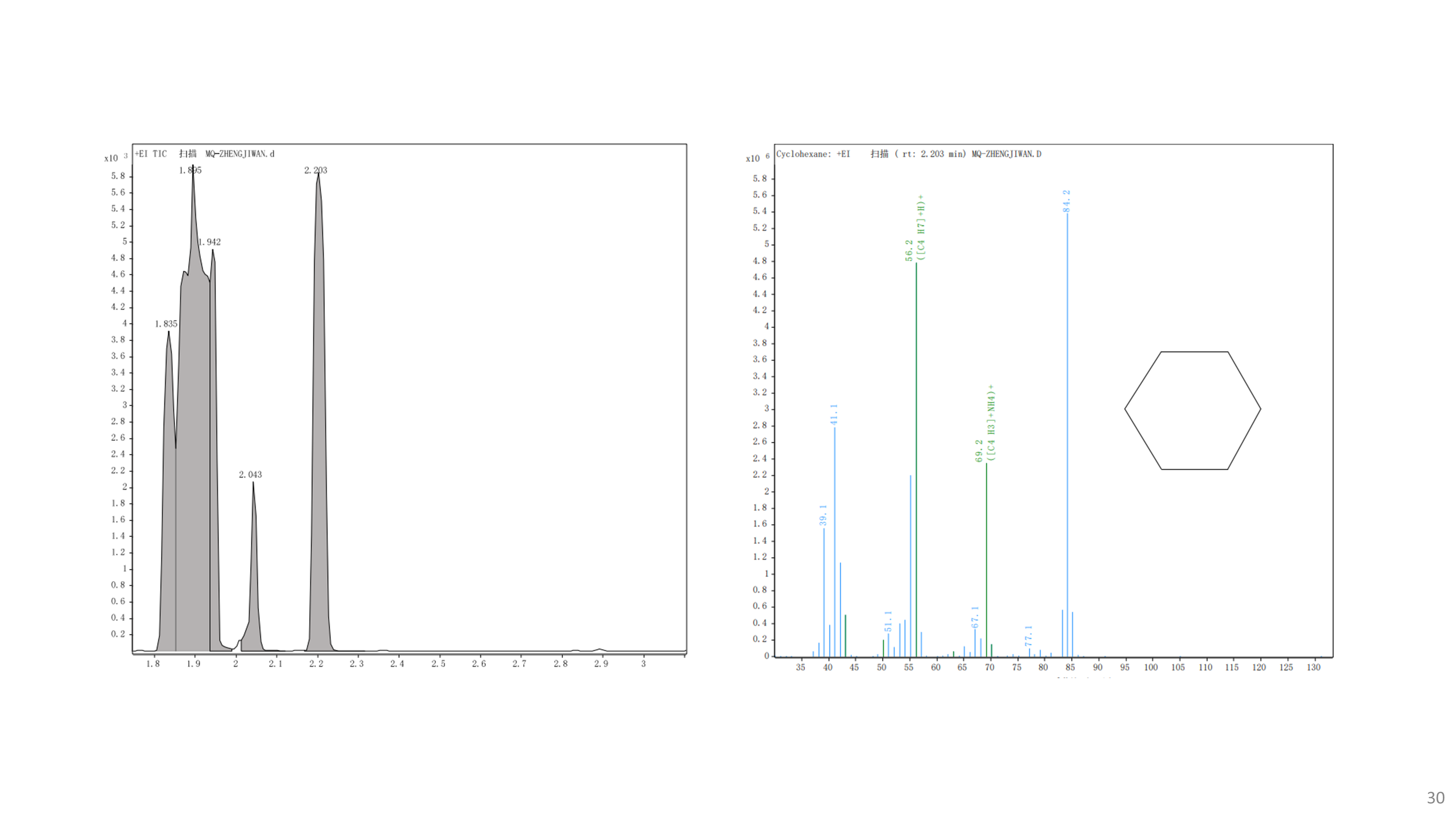
**

**Supplementary Fig. S19** The n-Hexane is converted to cyclohexane after light irradiation (λ = 254–365 nm).

In a nitrogen atmosphere, n-hexane undergoes irradiation by a mercury lamp, resulting in the generation of hydrogen gas in the reaction system. Additionally, analysis of the organic liquid by GC-MS revealed the formation of cyclohexane, with a retention time of 2.203 minute (**Fig. S19**). The experimental conditions were n-hexane (5 mL), consequent N_2_ (1 atm) at 20℃, irradiation of a Mercury lamp (λ= 254–365 nm), reaction time 5 h.

# Figure S20

**Supplementary Fig. S20** Construction of a closed-loop process.

Benzyl compounds can photosynthesize hydrogen peroxide (H_2_O_2_), which has the potential for for large-scale production of H_2_O_2_. However, there is an inevitable issue in this process, which is the handling of the dehydrogenation product of the benzyl substrate. Therefore, it makes sense to convert those dehydrogenation products back to the benzyl substrate to create a closed-loop process, and direct hydrogenation of those is a simple method to achieve this goal (**Fig. S20**).

Chen et al. reported a method using Pd_1_-mpg-C_3_N_4_^1^ as a catalyst to hydrogenate unsaturated bonds with water (H_2_O) as the hydrogen source. In this process, first, add 10 mg of Pd_1_-mpg-C_3_N_4_ and 2 mL of ultrapure water into the reactor, then sonicate for 5 minutes. Next, add 3 mL of 1,4-dioxane, 0.15 mmol of benzaldehyde (BD), and 0.5 mL of triethanolamine (TEOA). Seal the reactor with purified N_2_. Finally, under a temperature of 308 K, irradiate the reaction mixture for 4 hours. The outcome of this procedure reveals that nearly all of the benzaldehyde has been successfully reduced to benzyl alcohol.

# Figure S21&S22


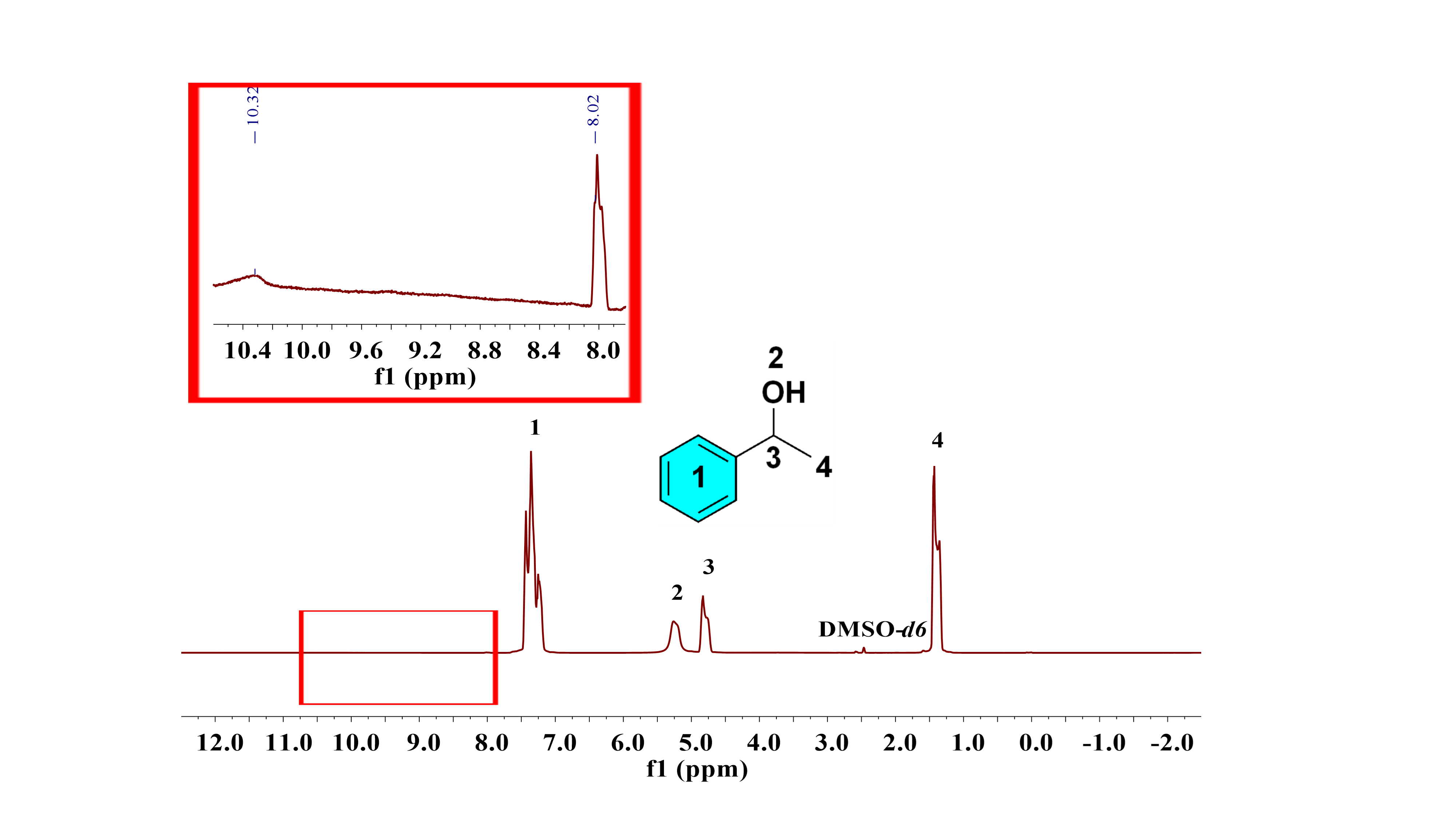


**Supplementary Fig. S21** The **^1^H NMR** of **H•** reduce CO_2_ to form HCOOH.


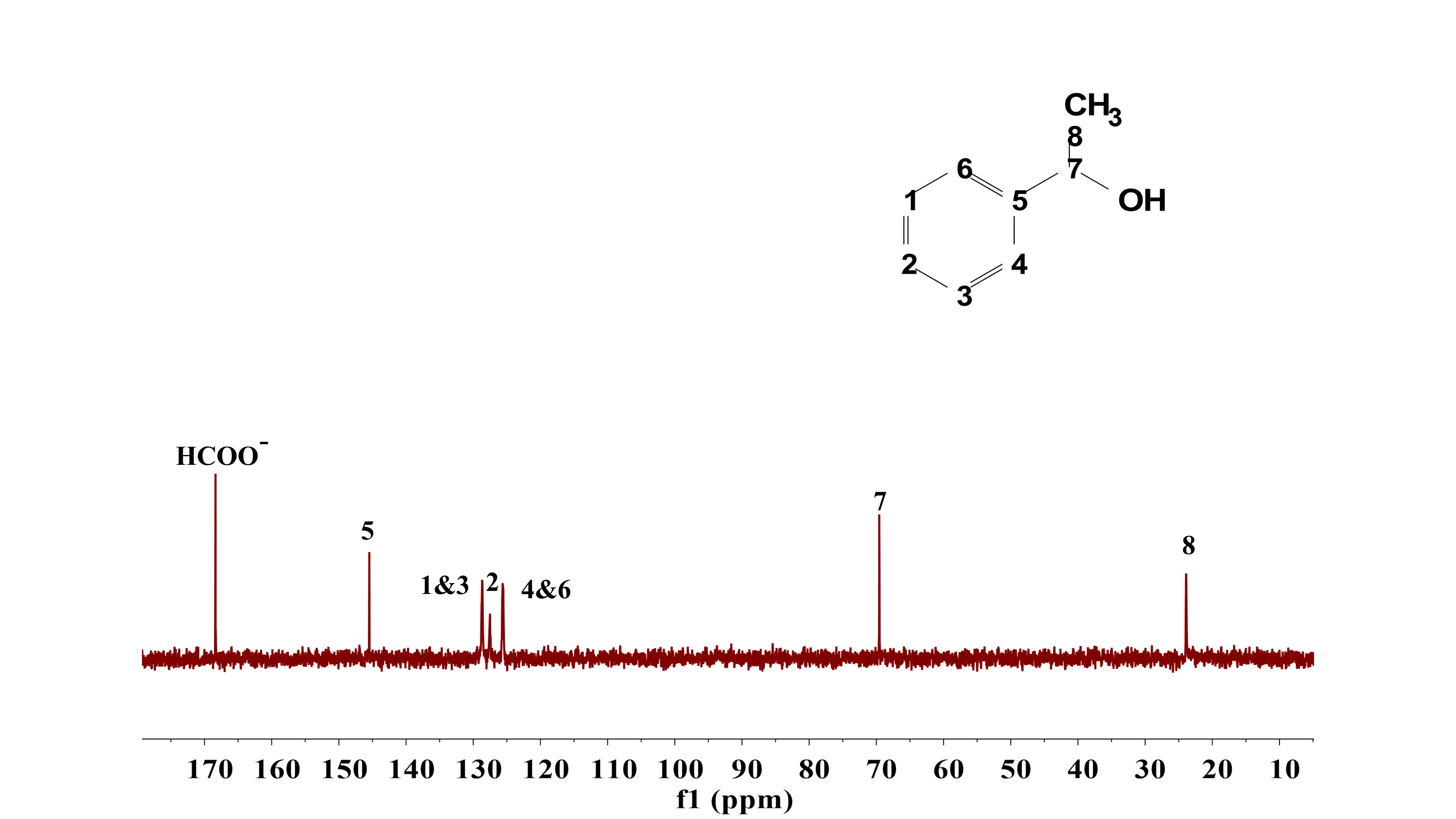


**Supplementary Fig. S22** The **^13^C NMR** of **H•** reduce CO_2_ to form HCOOH.

As shown in **Fig. S21**, the peaks of the active hydrogen of HCOOH were found in PA, **^1^H NMR (DMSO-*d_6_*, 400 MHz)**: δ = 10.34 (s, 1H), 7.96 (s, 1H). Then add **NaOH** and D_2_O to the PA reaction system, and detect the samples in the D_2_O by **^13^C NMR**, the carbonyl carbon peak of HCOO^-^ was discovered (as shown in **Fig. S22**).

# Figure S23

**Supplementary Fig. S23** The standard curve for H_2_.

As shown in **Fig. S23**, gas chromatography (GC) was used to record peak areas at H_2_ content of 0, 0.01%, 0.5%, and 1.5% in argon. A linear regression equation was then constructed to quantify the H_2_ content in an unknown sample based on the signal value. The H_2_ test method: 1 ml of gas was taken and fed into a gas chromatograph (FULI GC9790II) equipped with a thermal conductivity detector (TCD) and a 5A molecular sieve column using argon (99.999%) as the carrier gas for analysis.

# Figure S24

**Supplementary Fig. S24** The standard curve for H_2_O_2_.

As shown in **Fig. S24**, the UV-Vis absorbance peaks of H_2_O_2_ were monitored at wavelengths of 400 nm for concentrations of 68.0786 mg/L, 77.8042 mg/L, 90.7715 mg/L, 108.9258 mg/L, 136.1573 mg/L, 181.5430 mg/L and 272.3145 mg/L. A linear regression equation was constructed, enabling quantification of the amount of H_2_O_2_ in unknown samples based on their signal values.

# Figure S25

**Supplementary Fig. S25** The standard curve for HCOO^-^.

As shown in **Fig. S25**, peak areas for formate ions (HCOO^-^) were recorded by ion chromatography (HPIC) at concentrations of 0, 0.03125 ppm, 0.625 ppm, 1.25 ppm, 2.5 ppm, 5 ppm, 10 ppm, and 20 ppm. Subsequently, a linear regression equation was constructed to quantify the HCOO^-^ content in unknown samples based on signal values, yielding a correlation coefficient of 0.9993.

# References

1. Bordwell, F. G.; Zhang, S.; Zhang, X.-M.; Liu, W.-Z., Homolytic Bond Dissociation Enthalpies of the Acidic H-A Bonds Caused by Proximate Substituents in Sets of Methyl Ketones, Carboxylic Esters, and Carboxamides Related to Changes in Ground State Energies. *Journal of the American Chemical Society* **1995,** *117* (27), 7092-7096.
2. Zhao, E.*, et al.* Transfer Hydrogenation with a Carbon-Nitride-Supported Palladium Single-Atom Photocatalyst and Water as a Proton Source. *Angewandte Chemie (International ed. in English)* **61**(2022).
